# The novel GSDMD inhibitor GI‐Y2 exerts antipyroptotic effects to reduce atherosclerosis

**DOI:** 10.1002/ctm2.70263

**Published:** 2025-03-05

**Authors:** Xiaoxi Fan, Zhenfeng Cheng, Ruiyin Shao, Keke Ye, Xudong Chen, Xueli Cai, Shanshan Dai, Zhixuan Tang, Si Shi, Wenyuan Zheng, Weijian Huang, Jibo Han, Bozhi Ye

**Affiliations:** ^1^ Department of Cardiology and The Key Laboratory of Cardiovascular Disease of Wenzhou the First Affiliated Hospital Wenzhou Medical University Wenzhou Zhejiang China; ^2^ Department of Cardiology The Second Affiliated Hospital of Jiaxing University Jiaxing Zhejiang China; ^3^ Huzhou Central Hospital Affiliated Central Hospital of Huzhou University Huzhou China; ^4^ The Key Laboratory of Emergency and Disaster Medicine of Wenzhou Department of Emergency The First Affiliated Hospital of Wenzhou Medical University Wenzhou Zhejiang China; ^5^ First School of Medicine Wenzhou Medical University Wenzhou Zhejiang China; ^6^ Key Laboratory of Precision Medicine For Atherosclerosis Disease of Zhejiang Province Department of Cardiology Affiliated First Hospital of Ningbo University Ningbo Zhejiang China

**Keywords:** atherosclerosis, gasdermin D, macrophage, pyroptosis

## Abstract

**Introduction:**

Gasdermin D (GSDMD) and the pyroptosis it mediates are importantly involved in cardiovascular diseases (CVDs). Identifying and developing new inhibitors of GSDMD could be a promising strategy for treating pyroptosis‐mediated diseases, such as atherosclerosis.

**Objectives:**

We aimed to develop new inhibitor of GSDMD in atherosclerosis, as well as clarify the mechanisms underlying this inhibiting effect.

**Methods:**

Surface plasmon resonance and pull‐down assay were used to identify the amino acid sites of GSDMD inhibited by GI‐Y2. A mouse model of atherosclerosis was established by feeding a high‐fat diet for 12 weeks. After treating mice with GI‐Y2 (10 or 20 mg/kg, *i.g*.), the lipid plaque area on the arterial intimal surface, lipid deposition, collagen deposition and pyroptosis levels in aortic root sections were evaluated. Additionally, further treatment of atherosclerotic mice with macrophage membrane‐encapsulated GI‐Y2 was conducted to enhance the targeting ability of GI‐Y2 to atherosclerotic plaques.

**Results:**

In this study, we confirmed GI‐Y2 as a novel inhibitor of GSDMD via structure‐based virtual screening and pharmacological validation. Mechanistically, GI‐Y2 directly interacts with the Arg10 residue of GSDMD and reduces the membrane binding of GSDMD‐N. Functionally, we revealed that GI‐Y2 inhibits the formation of atherosclerotic plaques by targeting GSDMD. Similarly, GI‐Y2 reduces pyroptosis and macrophage infiltration in atherosclerosis. Furthermore, we constructed macrophage membrane‐coated GI‐Y2 nanoparticles to enhance the targeting of GI‐Y2 to macrophages in atheromatous plaques and demonstrated its vascular protective effect in vivo.

**Conclusion:**

This work demonstrated that GI‐Y2 can potentially alleviate CVDs by targeting GSDMD and provided a new compound for the study of GSDMD‐mediated pyroptosis.

**Key points:**

- We preliminarily confirmed GI‐Y2 as a novel inhibitor of GSDMD via structure‐based virtual screening and pharmacological validation.
- GI‐Y2 directly interacts with GSDMD and reduces the membrane binding of GSDMD‐N via the Arg10 residue.
- GI‐Y2 inhibits the formation of atherosclerotic plaques by targeting GSDMD and GI‐Y2 reduces pyroptosis and macrophage infiltration in atherosclerosis.
- We constructed macrophage membrane‐coated GI‐Y2 nanoparticles to enhance the targeting of GI‐Y2 to macrophages in atheromatous plaques and demonstrated its vascular protective effect in vivo.

## Graphical abstract

We confirmed GI‐Y2 as a novel inhibitor of GSDMD. GI‐Y2 directly interacts with the Arg10 residue of GSDMD and reduces the membrane binding of GSDMD‐N. We constructed macrophage membrane‐coated GI‐Y2 nanoparticles to enhance the targeting of GI‐Y2 to macrophages in atheromatous plaques and demonstrated its vascular protective effect.

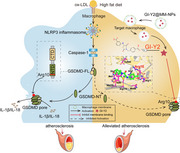

## 1 INTRODUCTION

Atherosclerosis, which is characterized as a chronic inflammatory condition impacting the arteries, is universally acknowledged as a significant factor in the progression of cardiovascular diseases (CVDs).^1^ Monocyte‐derived macrophages are pivotal inflammatory cells of atherosclerosis, occupying a crucial position throughout its various stages, encompassing both the initial plaque formation and eventual rupture.^2^ Pyroptosis represents a type of programmed cell death (PCD) closely related to inflammation and mediates the development of atherosclerosis.^3^ In animal models of atherosclerosis, macrophage pyroptosis is closely linked to the development and instability of plaques in atherosclerosis.^4^ Therefore, increasing attention has been given to macrophage pyroptosis in atherosclerosis.

Gasdermin‐D (GSDMD), a key protein in the progression of pyroptosis, was first discovered and reported in 2015.^5^, ^6^ Activated inflammatory caspases cleave GSDMD, releasing an N‐terminal domain (GSDMD‐N) that exhibits pore‐forming activity.^5^, ^6^The GSDMD‐N protein undergoes oligomerization on the cell membrane, creating pores that facilitate the release of inflammatory cytokines from the cytoplasm, thereby intensifying the inflammatory response.^5^, ^6^ Our previous studies showed that the key role of GSDMD in a variety of CVDs, including septic myocardial dysfunction,^7^ doxorubicin‐induced cardiotoxicity,^8^ cardiac hypertrophy,^9^, ^10^ vascular remodelling,^11^ abdominal aortic aneurysm^12^ and arteriosclerosis.^13^Numerous studies have reported that GSDMD is involved in the formation of atherosclerotic plaque.^14^, ^15^, ^16^, ^17^ In arteriosclerosis, we found that GSDMD is principally expressed in atherosclerotic macrophages and that macrophage‐derived GSDMD promotes aortic pyroptosis and atherosclerotic plaque formation in vivo,^13^ suggesting that GSDMD holds significant potential as a drug target for the effective treatment of atherosclerosis.

The development of GSDMD inhibitors holds promising prospects. GSDMD is a downstream regulatory factor of pyroptosis. The inhibition of GSDMD can reduce pyroptosis induced by several inflammasome components, such as NLPR3 and inflammatory caspases,^5^, ^6^ and that pharmacological inhibition of GSDMD exerts protective effects on atherosclerosis.^18^ Moreover, inhibiting the aggregation and perforation of GSDMD‐N on the cytomembrane could block the release of various pyroptotic molecules, such as IL‐1β and IL‐18.^5^, ^6^ In our previous study, we identified GI‐Y1 as an inhibitor of GSDMD via structure‐based virtual screening and revealed the inhibitory activity of GI‐Y1 in cardiac pyroptosis and myocardial ischemia/reperfusion injury.^19^ In this study, we optimized the virtual screening method and screened out GI‐Y2 as another potential candidate.

Here, we confirmed GI‐Y2 as a potential inhibitor of GSDMD via pharmacological validation. Mechanistically, GI‐Y2 directly interacts with GSDMD and specifically targets the Arg10 residue to attenuate the membrane‐binding capacity of GSDMD‐N. Functionally, we revealed that GI‐Y2 inhibits atherosclerotic plaque formation by targeting GSDMD. Similarly, GI‐Y2 reduces pyroptosis and macrophage infiltration in atherosclerosis. Furthermore, we constructed macrophage membrane‐coated GI‐Y2 nanoparticles to enhance the targeting of GI‐Y2 to macrophages in atheromatous plaques and demonstrated its vascular protective effect in vivo. This work demonstrated that GI‐Y2 can potentially alleviate atherosclerosis by targeting GSDMD and provided a new inhibitor for the study of GSDMD‐mediated pyroptosis.

## 2 MATERIALS AND METHODS

### 2.1 Structure‐based virtual screening

We previously screened 7 commercial compound libraries (approximately 5.5 million compounds) using a modelled GSDMD‐N as the template and identified 30 potential candidate compounds.^19^ In this study, we screened these candidate compounds using the crystal structure of GSDMD pore as a template. The crystal structure of GSDMD pore (PDB code 6VFE) was obtained from the PDB (http://www.rcsb.org/pdb/) and imported into Schrödinger Maestro (Schrödinger Release 2020–2: Maestro, Schrödinger, LLC, 2020). The structure was prepared using the “Protein Preparation Wizard” to ensure its structural correctness. This preparation included adding missing hydrogen atoms, assigning correct bond orders, correcting metal ionization states, and optimizing the hydrogen bond network. The prepared protein structure was then minimized to relieve any steric clashes and ensure a stable starting conformation. Small‐molecule compounds were prepared using “LigPrep”, which involves generating low‐energy conformations, assigning appropriate protonation states at physiological pH, and optimizing the geometry of the ligands. A receptor grid was generated in the protein structure using the default parameters in “Receptor Grid Generation”, defining the region of the protein where the ligands would be docked. The ligands were docked into the receptor grid using extra precision (XP) mode. The docked poses were evaluated by docking score, which combines various energy terms to predict binding affinities.

### 2.2 Modelling of the GSDMD‐GI‐Y2 complex

To enhance the precision and accuracy of predicting the potential binding sites of GSDMD for small molecules, the fpocket program was employed.^20^ Then, the *AutoDockFR 1.0* package was applied to predict the binding modes between GSDMD and GI‐Y2.^21^ A grid box of 25 Å × 25 Å × 25 Å was employed to cover the whole GSDMD binding site, and affinity maps were generated using the *agfr* module in the *AutoDockFR 1.0* package.^21^ Two hundred conformations were generated from the *adfr* module in the *AutoDockFR 1.0* package, and the optimal conformation was applied to molecular dynamics simulation studies.

The protocol for molecular dynamics simulations includes calculating the electrostatic potentials for GI‐Y2, aligning the force fields for the GSDMD‐GI‐Y2 complex, adding water molecules and appropriate counterions to the GSDMD‐GI‐Y2 complex, system minimizations, and heating and relaxing the system. Finally, 300 ns of MD simulations were performed, and trajectories from 200 to 300 ns with a total of 1,000 frames were applied for per‐residue binding free energy decomposition according to the molecular mechanics/generalized born surface area (MM/GBSA) method.^22^, ^23^

### 2.3 GI‐Y2‐GSDMD binding assays

The research involving human tissue samples was sanctioned by the Ethics Committee of the First Affiliated Hospital of Wenzhou Medical University (Approval No. KY2023‐053) and adheres to the guidelines set forth in the Declaration of Helsinki. Prior to the collection, written consent was obtained from the participants.

GI‐Y2 was purchased from SPECS. HUABIO successfully synthesized GST‐labelled GSDMD wild‐type protein (GST‐GSDMD) and GSDMD mutant protein (mutation of arginine to alanine at Arg10, GST‐GSDMD^R10A^). Pronase (10165921001, Sigma‐Aldrich) was used. Drug Affinity Responsive Target Stability (DARTS) assay was executed following the protocol outlined in a previously published research study.^24^

To perform the pull‐down assay, the streptavidin‐agarose beads (BeaverBeadsTM) were incubated with either GI‐Y2 or Bio‐GI‐Y2 (room temperature, 2 h). Untreated beads (Blank), Biotin and GI‐Y2 were used as controls. GSDMD protein, cell lysates and tissue lysates were subsequently incubated with beads coated with Bio‐GI‐Y2. The mixture underwent gently shaken (room temperature, 6 h), followed by three consecutive washes with PBS. The eluent was boiled with 5X loading buffer, and the sample was loaded onto a polyacrylamide gel for the purpose of western blot analysis. Total lysate was served as an input control.

To detect GI‐Y2 binding to GSDMD, a surface plasmon resonance (SPR) assay was performed. According to the instrument operation instructions, GST‐GSDMD was fixed on the chip surface, and then the GI‐Y2 compound was dissolved in DMSO. GI‐Y2 in PBS containing 1% DMSO was placed on the chip surface. During the dissociation process of the binding between GST‐GSDMD and GI‐Y2 in the flow path, the instrument detected changes in the resonance angle (SPR angle), from which the process curve and dissociation constant (*K_D_*) between molecules were obtained.

### 2.4 Cell culture

PMA was obtained from MedChemExpress (HY‐18739). Ox‐LDL was purchased from Yiyuan Biotechnology (YB‐002). Lipopolysaccharide (LPS, E. coli O111:B4 strain) was purchased from Sigma Aldrich. Nigericin (HY‐100381) was obtained from MedChemExpress. Human M‐CSF Recombinant Protein was obtained from PeproTech (300‐25‐2UG). We collected human peripheral blood and isolated peripheral blood mononuclear cells (PBMCs) with human whole blood mononuclear cell separation medium (tbdscience). Then we obtained monocytes through its adhesion property, which were subsequently induced into macrophages using human M‐CSF recombinant protein. The experiments utilizing human samples received ethical approval from the Ethics Committee of the First Affiliated Hospital of Wenzhou Medical University (Approval number KY2023‐053) and are conducted in accordance with the principles stipulated by the Declaration of Helsinki. The THP‐1 human monocyte leukaemia cell line, AC16 human cardiomyocyte cell line, HepG2 Human hepatocyte cell line and HEK‐293T human embryonic kidney cell line were obtained from the Shanghai Institute of Biochemistry and Cell Biology. Mouse primary peritoneal macrophages (MPMs) were washed down from the peritoneal cavity of *ApoE* ^−/‐^ mice as previously described.^25^ MPMs and THP‐1 cells were cultured in completed RPMI‐1640 medium (Gibco). AC16, HepG2, and HEK‐293T cells were cultured in completed DMEM (Gibco). The expression plasmids for Flag‐GSDMD, Flag‐GSDMD^R10A^ (mouse) and Flag‐GSDMD^R11A^ (mouse) were constructed by GeneChem Co., Ltd. The transfection of the GSDMD plasmid was executed utilizing LipofectAMINE 3000 (Thermo Fisher).

### 2.5 Preparation of macrophage membrane‐coated GI‐Y2 nanoparticles

PLGA (poly (lactic‐co‐glycolic acid), 50/50) was purchased from QIYUE Biology. GI‐Y2 powder (1 mg) was dissolved in 20 µL of DMSO and mixed with 10 mg of PLGA polymer dissolved in 1 mL of acetone. Then, the mixture was slowly added to 4 mL of distilled water. This mixture was stirred using a magnetic stirrer (IKA, Germany) at 1500 rpm for 5 min, after which the acetone in the emulsion was allowed to evaporate for 24 h. Thus, we obtained GI‐Y2@NPs. After ultrasonication, RAW264.7 cell membranes were isolated by multiple rounds of centrifugation with membrane extraction buffer. An ultrasound bath was used to wrap the macrophage membrane on the GI‐Y2@NP microspheres, resulting in macrophage membrane‐coated GI‐Y2 nanoparticles (GI‐Y2@MM‐NPs). The resulting GI‐Y2@MM‐NPs were freeze‐dried with a freeze dryer (Christ) for determination of the drug concentration using a full‐wavelength ultraviolet spectrophotometer (Implen NanoPhotometer, N50). The NP size (diameter, nm) was determined by a laser nanometre particle size analyser (Zetasizer Nano ZS, Malvern). The concentration of GI‐Y2 in GI‐Y2@MM‐NPs for injection was 69.52 ± 11.45 (± SD) mg/L. GI‐Y2 at the same concentration was used as a drug control.

### 2.6 Animal experiment

*Gsdmd* gene knockout mice on a C57BL/6J background (*Gsdmd* ^−/−^, strain no. T010437) and their littermates were purchased from GemPharmatech. *Gsdmd^−/‐^ ApoE^−/−^* mice were obtained as previous described.^13^ In compliance with the ethical guidelines set forth by the Laboratory Animal Ethics Committee (approval document no. WYYY‐IACUC‐AEC‐2024‐055), mice were maintained in the Animal Experiment Center of the First Affiliated Hospital of Wenzhou Medical University.

1. Sepsis‐related pyroptosis model. After the mice on the C57BL/6 background were given GI‐Y1 (20 mg/kg, *i.g*.), GI‐Y2 (20 mg/kg, *i.g*.), or an equivalent volume of vehicle (CMC‐Na, *i.g*.), sepsis was induced by administering 10 mg/kg lipopolysaccharide (LPS, *i.p*.) for 24 h. Subsequently, a second injection of either GI‐Y1 (20 mg/kg, *i.g*.) or GI‐Y2 (20 mg/kg, *i.g*.) was given to the mice in the respective groups. Mice in the LPS groups received an equivalent volume of vehicle (CMC‐Na, *i.g*.) as an injection. The survival rate of the mice was then observed and recorded over a period of 96 h, followed by survival analysis.
2. Atherosclerosis model. *ApoE^−/−^* Mice on the C57BL/6 background or *Gsdmd^−/‐^ ApoE^−/−^* Mice were administered a high‐fat diet (HFD) for a duration of 12 weeks to induce atherosclerosis. The control mice were fed a normal low‐fat diet (LFD). For treatment with the inhibitor, after a 6‐week HFD, the mice were given GI‐Y2 (10 or 20 mg/kg/2 day, dissolved in 1% CMC‐Na, *i.g*.) and then a 6‐week HFD. For GI‐Y2@MM‐NP treatment, mice were administered a HFD for a duration of 8 weeks before tail vein injection of GI‐Y2@MM‐NPs (0.03 mg/kg/3 days, *i.v*.). The GI‐Y2 group was given the same concentration of GI‐Y2 intravenously (0.03 mg/kg/3 days). After these experiments were completed, the mice were humanely euthanized using sodium pentobarbital anaesthesia.

### 2.7 Analysis of atherosclerotic lesions

The entire aorta (aortic arch, thoracic and abdominal segments) was longitudinally cut and stained with Oil Red O solution. Images were taken, and the number of atherosclerotic plaques on the aortic artery surface was counted. Part of the heart tissue containing the root was embedded in OCT (Sakura) compound, ensuring that three valve cusps were visible during the frozen tissue sectioning process. The stained sections of the aortic root were taken from the sections 10–30 µm after the first appearance of the aortic valve. Three to six sections from each group were subjected to Oil Red O staining, Masson's trichrome staining, and immunohistochemistry, and 6–8 microscope fields were randomly selected by blinded experimenters for analysis. The lesion area was quantified using ImageJ software, which reflects the lesion size or expression level of a specific section rather than the average of multiple sections.

### 2.8 Immunostaining

The proximal aorta was stained with HE, Oil Red O and Masson's trichrome (Solarbio). For immunofluorescence, primary antibodies against GSDMD (ab219800, Abcam, 1:200) and F4/80 (sc‐26642, Santa Cruz, 1:200) were used, followed by incubation with Alexa‐488/647 (Abcam, 1:5000) and DAPI. For immunohistochemical staining, primary antibodies against F4/80 (sc‐26642, Santa Cruz, 1:200), Ly6C (HA500088, Abcam, 1:200) and Ly6G (0809‐11, Abcam, 1:200) were used. Subsequently, the sections underwent incubation with secondary antibodies, diaminobenzidine and hematoxylin.

### 2.9 Serum biochemical analysis and analysis of cell supernatants

After the mice were euthanized, blood was collected and centrifuged at 2000 × g for 10 min to obtain plasma samples. Total cholesterol, triglyceride and LDL‐cholesterol levels in the serum were detected by a Beckman AU480 analyzer. Lactate dehydrogenase levels in the cell supernatant were detected with an assay kit (BC0685, Solarbio) according to the manufacturer's instructions. IL‐1β and IL‐18 levels were measured by ELISA kits (eBiosciences). Cell Counting Kit‐8 (CCK‐8, E‐CK‐A362, Elabscience) was employed as a quantitative measurement to evaluate the vitality of the cells (%). LDH Cytotoxicity Assay Kit (C0017, Beyotime Biotechnology) was employed to evaluate the cell mortality (%).

### 2.10 Real‐time quantitative polymerase chain reaction (RT‐qPCR)

TRIzol reagent (Thermo Fisher) was used to extract total RNA from cellular or aortic tissues. cDNA synthesis was achieved through reverse transcription reactions with PrimeScript RT reagent kits (Thermo Fisher). Quantitative real‐time polymerase chain reaction (RT‐qPCR) was conducted on an Applied Biosystems 7300 system with SYBR Green reagent kits (TaKaRa). The primer sequences utilized in this study are presented in Table S1.

### 2.11 Cell membrane protein extraction

We extracted cell membrane protein with cell membrane protein and cytoplasmic protein extraction kit (P0033, beyotime). The present kit employs a homogenization process to partially disrupt cells. Subsequently, low‐speed centrifugation is utilized to remove the nuclei and a small number of intact cells, which form the pellet. The supernatant is then subjected to high‐speed centrifugation to obtain the membrane pellet and the supernatant containing cytoplasmic proteins. Finally, an optimized membrane protein extraction reagent is applied to the pellet to extract membrane proteins.

### 2.12 Western blot analysis

Primary antibodies against Na‐K‐ATPase (1:1000, 3010S) were obtained from CST. Antibodies against IL‐1β (ab9722, 1:1000) and GSDMD (ab219800, 1:1000) were obtained from Abcam. Antibodies against IL‐18 (10663‐1‐AP, 1:1000) and Flag (1:1000, 20543‐1‐AP) were obtained from Proteintech. GAPDH (1:10000, MB001) was obtained from Bioworld. Western blotting was performed as previously described.^8^, ^9^

### 2.13 Electron microscopy and image processing

Transfer GI‐Y2@MM‐NPs (10 µL) onto the carbon support film of the electron microscopy grid and stain with 2% uranyl acetate. Imaging of GI‐Y2@MM‐NPs was performed using a HITACHI HT7800 electron microscope at 80 kV. The magnification of the images is 80,000×.

### 2.14 Statistical analysis

To ensure unbiased evaluation, the data underwent a blinded analysis. Additionally, GraphPad Prism 8 was employed as the tool for conducting the statistical analysis. The selection of appropriate data processing methods is based on the normality test of the data. Normality is determined by the Shapiro–Wilk test. If the data is normally distributed, the Student's *t*‐test is used for two groups; for more than two groups, one‐way ANOVA with Bonferroni correction is applied. If the data is not normally distributed, the Mann–Whitney test is used for two groups; for more than two groups, the Kruskal–Wallis test with Dunn's multiple comparison is applied. For groups of three or more, the statistical method of one‐way ANOVA was employed, along with a post hoc analysis incorporating the Bonferroni correction to ensure the validity and reliability of the results. To compare the differences between two groups, the Student's *t*‐test was employed. A *p*‐value < 0.05 was considered to indicate statistical significance.

## 3 RESULTS

### 3.1 Structure‐based virtual screening and pharmacological validation identified GI‐Y2 as a novel GSDMD inhibitor

**FIGURE 1:**
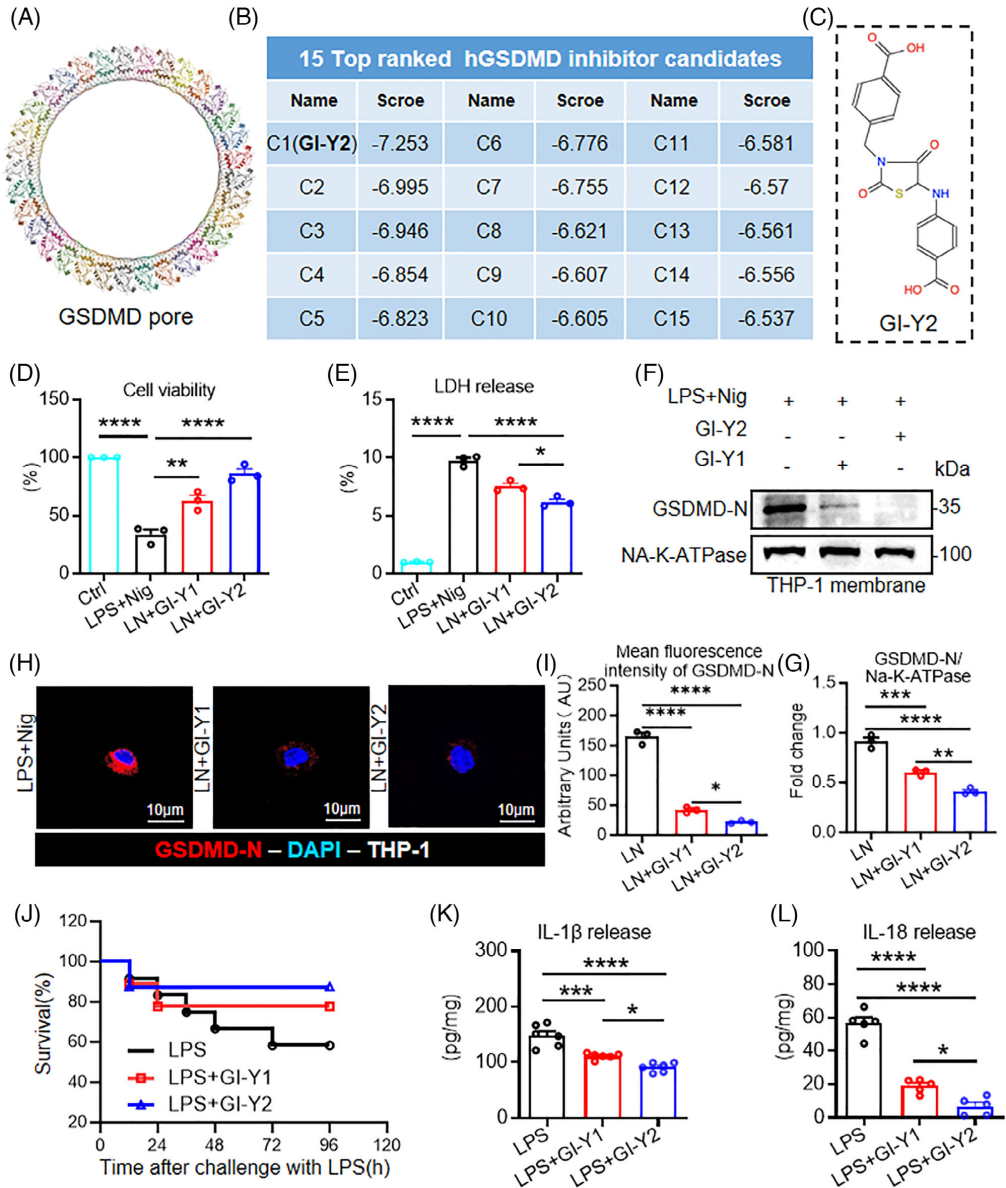
Structure‐based virtual screening and pharmacological validation identified GI‐Y2 as a novel gasdermin D (GSDMD) inhibitor. (A) The crystal structure of GSDMD pore (PDB: 6VFE). (B) Fifteen top‐ranked candidate compounds identified by structure‐based virtual screening. (C) Chemical structural formula of GI‐Y2. (D–I) THP‐1 cells stimulated with PMA were pretreated with GI‐Y2 (20 µM, 1 h) or GI‐Y1 (20 µM, 1 h) and then stimulated with LPS+nigericin (LN; 1 µg/ml, 4 h for LPS; 10 µM, 30 min for nigericin) (*n* = 3). Cell viability (D) and LDH release (E) were detected. Expression of GSDMD‐N in the THP‐1 cell membrane (F) and quantification (G). Immunofluorescence staining of GSDMD‐N (H) and quantification of the mean fluorescence intensity of GSDMD‐N (I). (J–L) Mice were pretreated with GI‐Y1 (20 mg/kg, *i.g*., twice), GI‐Y2 (20 mg/kg, *i.g*., twice) or the control vehicle before LPS stimulation (10 mg/kg, i.p., once, 24 h). The survival curves of the mice were recorded (n = 10) (J), and the serum IL‐1β (K) and IL‐18 (L) levels in the mice were detected (*n* = 6). * *p* < 0.05, ** *p* < 0.01, *** *p *< 0.001, **** *p* < 0.0001.

In our previous research, we identified a novel GSDMD inhibitor, GI‐Y1, via structure‐based virtual screening.^19^ In addition, we discovered that GI‐Y1 possesses the capability to hinder the pyroptotic pore formation of GSDMD‐N to reduce pyroptosis.^19^ In this study, we improved the structure‐based virtual screening by using the crystal structure of the GSDMD pore (Figure 1A, PDB: 6VFE^26^) as a template instead of a modelled GSDMD‐N. We previously screened 7 commercial compound libraries (approximately 5.5 million compounds) using a modelled GSDMD‐N as the template and identified 30 potential candidate compounds.^19^ In this study, we screened these candidate compounds using the crystal structure of GSDMD pore as the template and found that GI‐Y2 had the highest score (Figure 1B,C).

We first validated the antipyroptotic effect of GI‐Y2 in an LPS+nigericin (LN)‐induced macrophage pyroptosis model. The classic macrophage pyroptosis model was successfully constructed in Tph‐1 cells (Figure S1A–C). GI‐Y2 exhibited greater inhibitory effects than GI‐Y1 in PMA‐differentiated THP‐1 cells, as evidenced by cell viability (Figure 1D) and LDH release (Figure 1E). Subsequently, we found that the binding between cytomembrane and GSDMD‐N could be effectively inhibited by both GI‐Y1 and GI‐Y2 (Figure 1F–I), indicating that GI‐Y2 might suppress pyroptosis by inhibiting the cytomembrane binding of GSDMD‐N and the IC50 is 35.40 ± 1.97 µM (Figure S1D). Finally, we verified the efficacy of GI‐Y2 in inhibiting GSDMD across various tissues, including human cardiomyocyte cell line AC16, human renal epithelial cell line 293T, human hepatocyte cell line HepG2 (Figure S1E–J).

We also investigated the biosafety of GI‐Y2 in vivo. Administration of GI‐Y2 did not affect the body weight, hematologic system, hepatic function, renal function or tissue structure of the brain, lung, liver, kidney or heart (Figure S2). We then examined the antipyroptotic effects of GI‐Y2 in a sepsis‐related pyroptosis mouse model. GI‐Y2 was more effective than GI‐Y1 at increasing the survival rate (Figure 1J) and reducing the serum IL‐1β and IL‐18 levels (Figure 1K,L) in septic mice. Overall, through structure‐based virtual screening and subsequent pharmacological validation, we identified GI‐Y2 as a GSDMD inhibitor that inhibits the binding between membrane with GSDMD‐N.

### 3.2 GI‐Y2 directly interacts with GSDMD and reduces the membrane binding of GSDMD‐N

**FIGURE 2:**
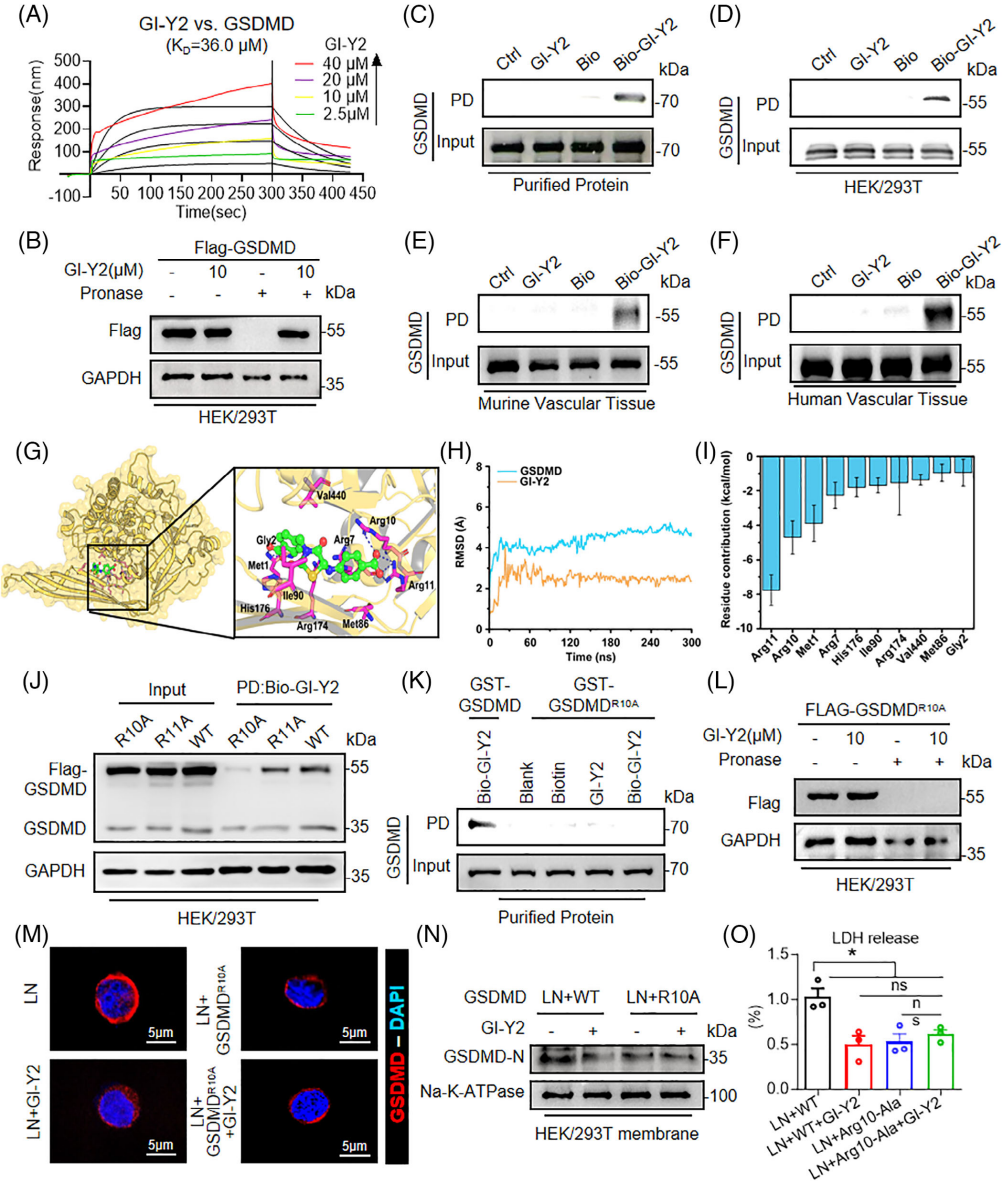
GI‐Y2 directly interacts with gasdermin D (GSDMD) and reduces the membrane binding of GSDMD‐N. (A) A surface plasmon resonance (SPR) was utilized to determine the binding affinity between GI‐Y2 and GSDMD (*K_D_* = 36.0 µM). (B) HEK/293T cells transfected with Flag‐GSDMD were treated with GI‐Y2 (10 µM) for 1 h and then subjected to DARTS. (C–F) The interaction between biotinylated GI‐Y2 (Bio‐GI‐Y2) and GI‐Y2 was detected by a pull‐down (PD) assay. GSDMD protein was obtained from purified GST‐GSDMD protein (C), cell lysates from HEK/293T expressing Flag‐GSDMD (D) and tissue lysates from mouse (E) or human (F) atherosclerotic vascular tissues. (G) Overview of GSDMD (yellow) interactions with GI‐Y2 (green). Zoom: detailed view of the 10 residues with the greatest contribution to GSDMD (yellow) interacting with GI‐Y2 (green) and hydrogen binding (blue). (H) RMSD curves of the backbone atoms of GSDMD and the heavy atoms of GI‐Y2 during the 300 ns MD simulation. (I) The top 10 residues of GSDMD bound to GI‐Y2. J‐K The interaction between Bio‐GI‐Y2 and mutant GSDMD (Arg10 or Arg11 residue mutation) was detected by pull‐down (PD) assays. GSDMD protein was obtained from the lysates of HEK/293T cells expressing Flag‐GSDMD^R10A^ or Flag‐GSDMD^R11A^ (J) and purified GST‐GSDMD^R10A^ protein (K). (L) HEK/293T cells transfected with Flag‐GSDMD^R10A^ were treated with GI‐Y2 (10 µM) for 1 h and then subjected to DARTS. (M–O) HEK/293T cells expressing Flag‐GSDMD^R10A^ were treated with GI‐Y2 (20 µM, 24 h) and then stimulated with LN (1 µg/ml, 4 h for LPS; 10 µM, 30 min for nigericin). Immunofluorescence staining of GSDMD‐N in HEK/293T membranes (M). Expression of GSDMD‐N in HEK/293T cell membranes (N). LDH release (O). (*n* = 3; * *p* < 0.05, ns *p* > 0.05).

To assess the direct association between GI‐Y2 and GSDMD, we conducted drug‐protein binding experiments, including a surface plasmon resonance (SPR) assay, a drug affinity responsive target stability (DARTS) assay and a pull‐down assay. The SPR assay showed direct binding of GI‐Y2 to the GSDMD protein (Figure 2A, *K_D_* value = 36.0 µM). Similarly, the DARTS assay confirmed the interaction between GI‐Y2 and GSDMD in HEK/293T expressing Flag‐GSDMD (Figure 2B). Next, GI‐Y2 was biotinylated (Bio‐GI‐Y2, Figure S3A) and subjected to a pull‐down experiment. As shown in Figure S3B–D, Bio‐GI‐Y2 retained its antipyroptotic activity. The pull‐down analysis revealed that Bio‐GI‐Y2 binds to GSDMD through the utilization of purified GST‐GSDMD protein (Figure 2C), cell lysis from HEK/293T cells expressing Flag‐GSDMD (Figure 2D) and tissue lysis from mouse (Figure 2E) or human (Figure 2F) atherosclerotic vascular tissues.

To determine the binding mode between GSDMD and GI‐Y2, we applied molecular docking (Figure 2G) and molecular dynamics (MD) simulations. The RMSD analysis revealed that GI‐Y2 and GSDMD reached dynamic stability after 150 ns of simulation (Figure 2H). Afterward, the stable trajectories from 200 to 300 ns were subjected to per‐residue binding free energy decomposition calculations. As shown in Figure 2I, the 10 residues with the greatest contributions are Arg11, Arg10, Met1, Arg7, His176, Ile90, Arg174, Val440, Met86 and Gly2. Structural analysis suggested that GI‐Y2 forms a total of 4 hydrogen bonds with Arg10 and Arg11 (Figure 2G).

To determine the binding residue of GI‐Y2, we constructed 2 mutants of GSDMD (mutation of arginine to alanine at Arg10 or Arg11, R10A or R11A). Pull‐down experiments showed that GI‐Y2 failed to interact with GSDMD^R11A^ but still interacted with GSDMD^R10A^ (Figure 2J), suggesting that Arg10 might be the crucial binding residue of GI‐Y2. Pull‐down with purified GST‐GSDMD protein (Figure 2K) and DARTS (Figure 2L) further confirmed that GI‐Y2 did not bind to GSDMD^R10A^. Notably, Arg10 is predicted to bind lipids and mediate the oligomerization of GSDMD‐N.^27^ Thus, we examined whether GI‐Y2 targets the Arg10 residue to inhibits the membrane binding of GSDMD‐N. As shown in Figure 2 M,N, Figure S3E,F, both GI‐Y2 and GSDMD^R10A^ significantly inhibited the membrane binding of GSDMD‐N, while the combination of GI‐Y2 and GSDMD^R10A^ showed no significant increase in their inhibitory effect. These results were further corroborated by the LDH release triggered by pyroptotic pore formation (Figure 2O). The above results indicate that GI‐Y2 directly interacts with GSDMD and targets the Arg10 residue to reduces the cytomembrane binding of GSDMD‐N.

### 3.3 GI‐Y2 exhibits a therapeutic effect on the formation of atherosclerotic plaques in vivo

**FIGURE 3:**
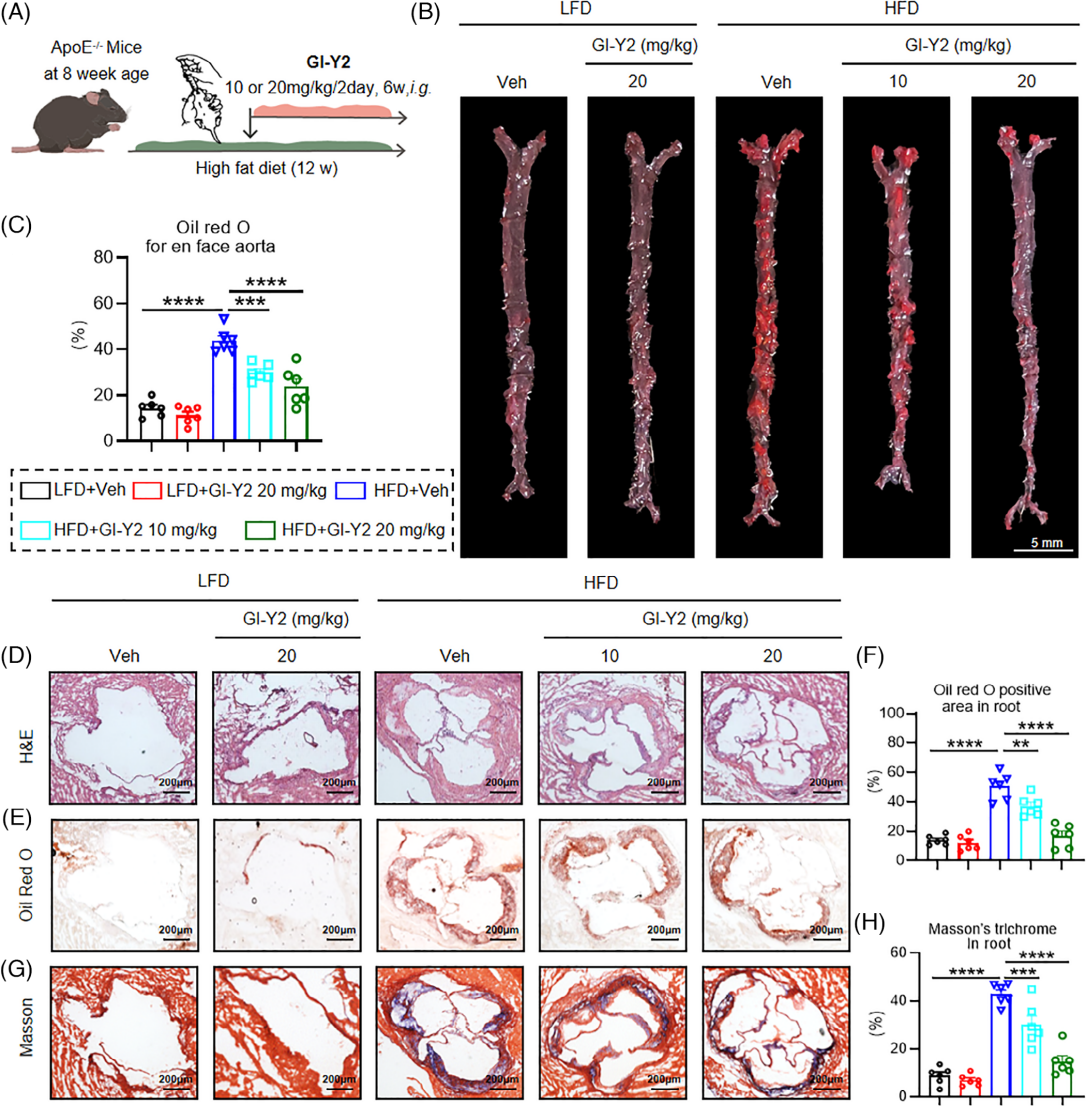
GI‐Y2 exhibits a therapeutic effect on the formation of atherosclerotic plaques in vivo. (A) *ApoE* ^−/−^ mice were fed a high‐fat diet (HFD) for 6 weeks, followed by intragastric administration of GI‐Y2 (10 or 20 mg/kg/2 day, i.g.) for an additional 6 weeks. (B, C) Oil Red O staining of the en face aorta (B) and quantification (C). (D) HE staining of the aortic root. (E, F) Oil Red O staining (E) and quantification of positive areas (F) of the aortic root. (G, H) Masson's trichrome staining (G) and quantification of fibrotic areas (H) in the aortic root. (*n* = 6, ** *p* < 0.01, *** *p* < 0.001, **** *p* < 0.0001).

To prove the therapeutic role of GI‐Y2 in atherosclerosis, *ApoE* ^−/−^ mice were fed a HFD for 6 weeks, followed by intragastric administration of GI‐Y2 (10 or 20 mg/kg/2 day, i.g.) for an additional 6 weeks (Figure 3A). We first validated the establishment of hyperlipemia in *ApoE* ^−/−^ mice induced by a HFD (Figure S4). As shown in Figure 3B,C, administration of GI‐Y2 dose‐dependently reduced atherosclerotic plaques in HFD‐fed *ApoE* ^−/−^ mice, as indicated by en face Oil Red O staining of the aortas. Similarly, a significant reduction in the lesion size of the aortic root was caused by treatment with GI‐Y2 in a dose‐dependent manner (Figure 3D–F). Similarly, GI‐Y2 exhibited dose‐dependent inhibitory effects on HFD‐induced aortic fibrosis in vivo (Figure 3G,H). These results demonstrated that GI‐Y2 has a therapeutic effect on HFD‐induced atherosclerotic plaque formation in *ApoE* ^−/−^ mice.

### 3.4 GI‐Y2 inhibits the formation of atherosclerotic plaques by targeting GSDMD

**FIGURE 4:**
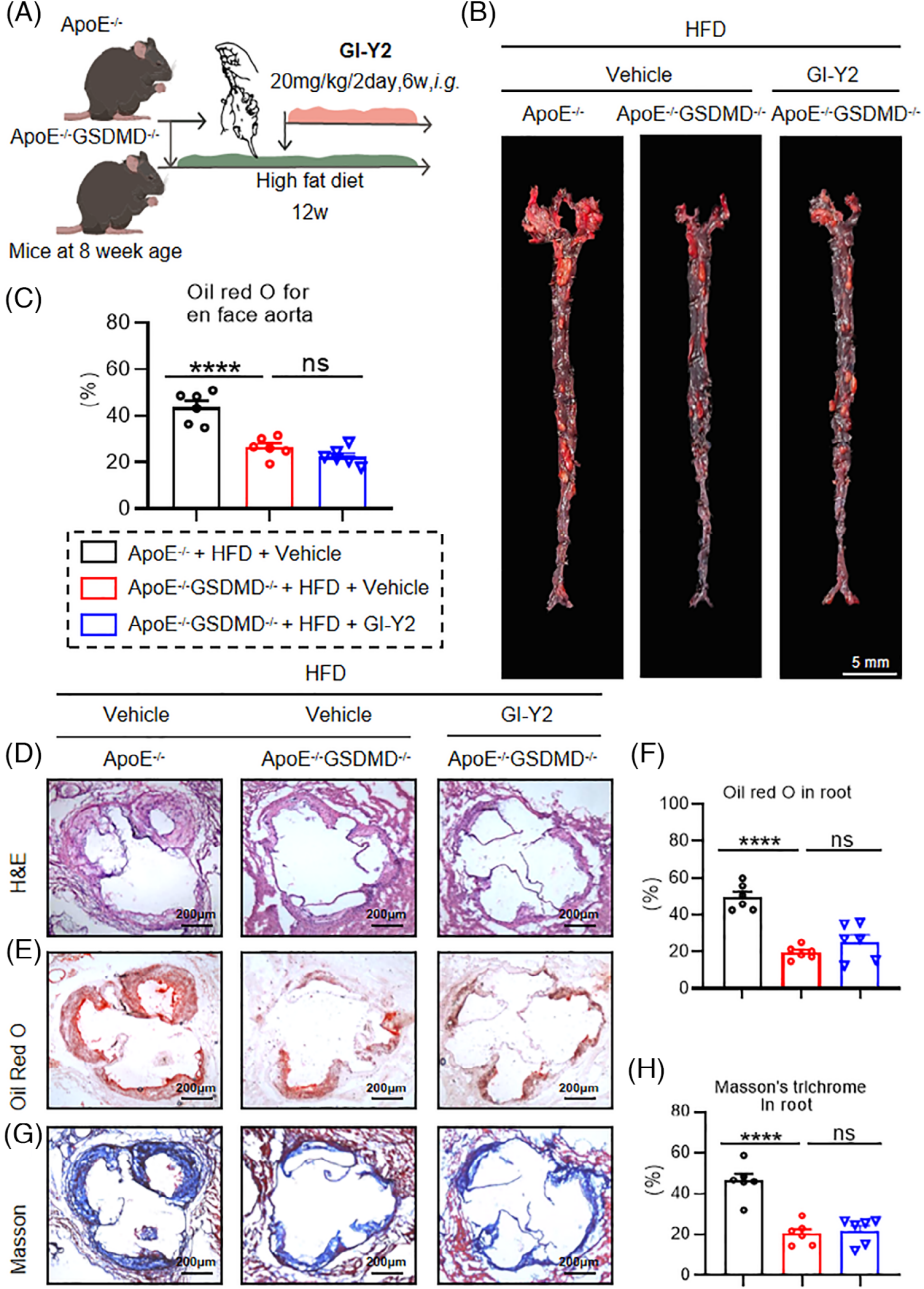
GI‐Y2 inhibits the formation of atherosclerotic plaques by targeting gasdermin D (GSDMD). (A) Schematic illustration showing the development of atherosclerosis in *ApoE* ^−/−^ and *ApoE* ^−/−^ GSDMD^−/−^ mice treated with GI‐Y2 (20 mg/kg/2 day, i.g.). (B, C) Oil Red O staining of the en face aortas of atherosclerotic mice (*ApoE* ^−/−^ and *ApoE* ^−/−^ GSDMD^−/−^) treated with GI‐Y2 (B) and quantification (C). (D) HE staining of the aortic root. (E, F) Oil Red O staining (E) of the aortic root and quantification of positive areas (F). (G, H) Masson's trichrome staining (G) and quantification of fibrotic areas (H). (*n* = 6, ns *p* > 0.05, **** *p* < 0.0001).

To examine the effect of GSDMD on GI‐Y2‐mediated vasoprotection, we examined the function of GI‐Y2 in atherosclerosis with *Gsdmd* deficiency (*Gsdmd^−/−^ApoE^−/−^*, Figure 4A). En face Oil Red O staining of aortas revealed that atherosclerotic lesions were reduced by *Gsdmd* deficiency (Figure 4B,C), while treatment with GI‐Y2 (20 mg/kg/2 day, i.g.) did not have a greater inhibitory effect on *Gsdmd^−/−^ApoE^−/−^* mice (Figure 4B,C), implying that the GI‐Y2‐mediated vasoprotective effect on atherosclerosis is achieved by targeting GSDMD. Similarly, the administration of GI‐Y2 did not further reduce the lesion area (Figure 4D), lipid deposition (Figure 4E,F) or interstitial fibrosis (Figure 4G,H) in the aortic roots of HFD‐induced *Gsdmd^−/−^ApoE^−/−^* mice. After the overexpression of GSDMD in macrophages, there was a notable increase in both membrane‐bound GSDMD‐N and the release of IL‐1β. Additionally, the mRNA levels of various inflammatory chemokines and adhesion molecules, including *Cxcl*, *Ccl2*, *Vcam1*, and *Icam1*, also exhibited significant elevation. GI‐Y2 mitigated these upward trend of these indicators (Figure S5A–G). These results showed that GI‐Y2 inhibited the formation of atherosclerotic plaques by targeting GSDMD.

### 3.5 GI‐Y2 reduces HFD‐induced aortic pyroptosis and macrophage infiltration in *ApoE^−/−^* mice

**FIGURE 5:**
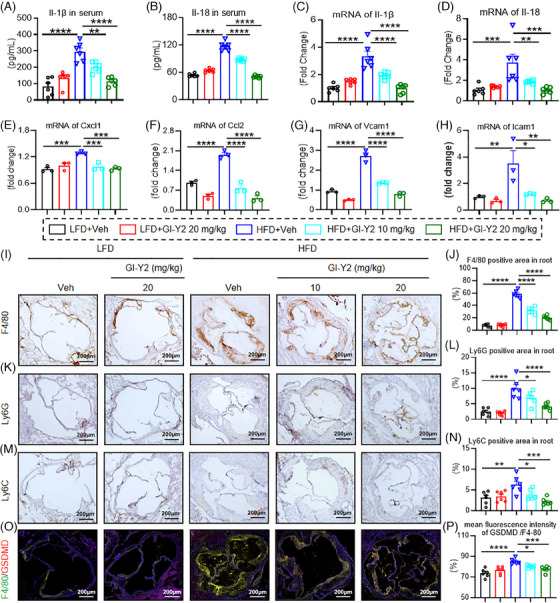
GI‐Y2 reduces high‐fat diet (HFD)‐induced aortic pyroptosis and macrophage infiltration in *ApoE^−/−^* mice. *ApoE* ^−/−^ mice were fed a HFD for 6 weeks, followed by intragastric administration of GI‐Y2 (10 or 20 mg/kg/2 day, i.g.) for an additional 6 weeks. (A, B) ELISA was used to measure the serum levels of IL‐1β (A) and IL‐18 (B). (C–H) The mRNA levels of *IL‐1β* (C), *IL‐18* (D), *Cxcl* (E)*, Ccl2* (F)*, Vcam1* (G), and *Icam1* (H) in the aortas of atherosclerotic mice were measured. (I, J) Immunohistochemical staining of F4/80 (I) was performed on the aortic root, and the positive areas were quantified (J). (K, L) Immunohistochemical staining of Ly6G (K) in the aortic root and quantification of Ly6G‐positive areas (L). M‐N Immunohistochemical staining of Ly6C (M) was performed on the aortic root, and the positive areas were quantified (N). O Representative immunofluorescence images showing the colocalization of F4/80 and GSDMD in aortic sections. (P) Quantification of the mean fluorescence intensity of GSDMD/F4‐80 (*n* = 6 for A–D, J, L, N and P; *n* = 3 for E–H; * *p* < 0.05 ** *p *< 0.01, *** *p *< 0.001, **** *p *< 0.0001).

To verify the antipyroptotic effect of GI‐Y2 in atherosclerotic mice, we first tested inflammatory factors in HFD‐fed *ApoE^−/−^* mice. Treatment with GI‐Y2 dose‐dependently reduced the serum levels of the pyroptosis‐related inflammatory cytokines IL‐1β and IL‐18 (Figure 5A,B). Similarly, GI‐Y2 also inhibited the mRNA levels of IL‐1β and IL‐18 in aortic tissues (Figure 5C,D). Similarly, the mRNA levels of other inflammatory chemokines and adhesion molecules in aortic tissues, such as Cxcl, Ccl2, Vcam1 and Icam1, were also reduced by GI‐Y2 in a dose‐dependent manner (Figure 5E–H). Our previous study showed that GSDMD mediated the infiltration and migration of macrophages during atherosclerosis.^13^ Here, administration of GI‐Y2 dose‐dependently reduced the infiltration of inflammatory cells, such as macrophages (Figure 5I,J, marked by F4/80), neutrophils (Figure 5K,L, marked by Ly6G) and monocytes (Figure 5 M,N, marked by Ly6C). Using single‐cell RNA sequencing, we previously confirmed that the expression of GSDMD is principally increased in macrophages in atherosclerosis.^13^ Here, double fluorescence staining verified these results, and further revealed that GI‐Y2 dose dependently reduced the expression of macrophage GSDMD in HFD‐fed *ApoE^−/−^* mice (Figure 5O,P). The above results indicated that GI‐Y2 reduces aortic pyroptosis and macrophage infiltration during atherosclerosis.

### 3.6 GI‐Y2 inhibits ox‐LDL‐induced macrophage pyroptosis in vitro

**FIGURE 6:**
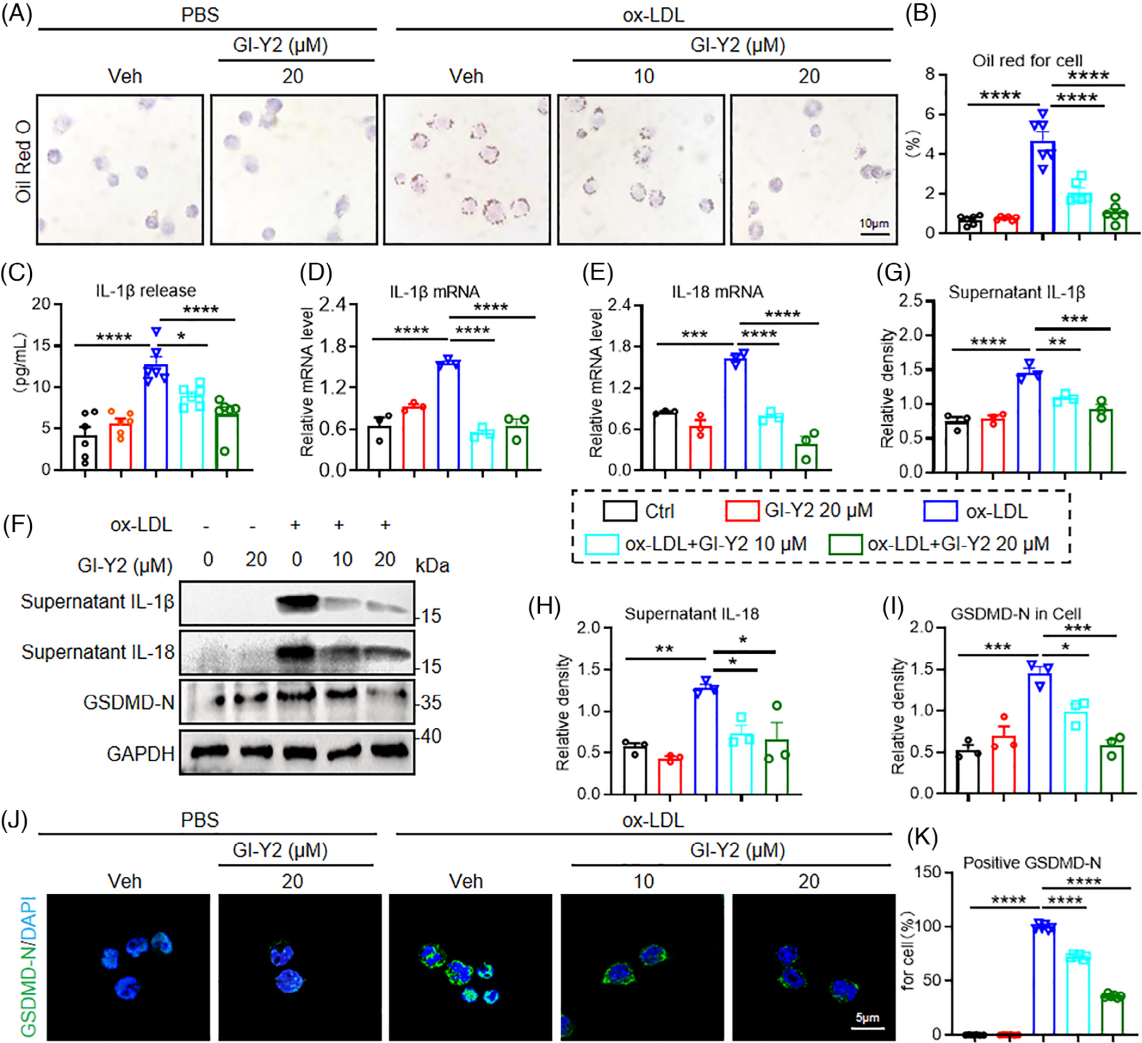
GI‐Y2 inhibits ox‐LDL‐induced macrophage pyroptosis in vitro. MPMs were pretreated with GI‐Y2 (10 or 20 µM, 1 h) or vehicle (DMSO, 1 h) and then stimulated with ox‐LDL (50 µg/mL, 24 h). (A, B) Oil Red O staining of ox‐LDL‐induced MPMs pretreated with GI‐Y2 (A) and quantification (B, *n* = 6). (C) ELISA was used to measure the release of IL‐1β in ox‐LDL‐treated MPMs (*n* = 6). (D, E) The mRNA levels of IL‐1β and IL‐18 (*n* = 3). F Immunoblot analysis of supernatant IL‐1β, supernatant IL‐18, and GSDMD‐N. G‐I. Densitometric quantification of immunoblot detection of supernatant IL‐1β (G), supernatant IL‐18 (H), and GSDMD‐N (I), in Figure 6F (*n* = 3). J‐K Representative immunofluorescence images of MPMs (J) and quantification (K, *n* = 6). * *p* < 0.05, ** *p *< 0.01, *** *p *< 0.001, **** *p* < 0.0001.

Monocyte‐derived macrophages, the key inflammatory cells involved in the process of atherosclerosis, play an important role in all stages, from plaque formation to plaque rupture.^28^ Oil Red O staining revealed that GI‐Y2 dose‐dependently inhibited the uptake/phagocytosis of ox‐LDL in MPMs (Figure 6A,B). Furthermore, we delved into the inhibitory effect of GI‐Y2 on ox‐LDL‐induced macrophage pyroptosis. As shown in Figure 6C, GI‐Y2 exhibited a dose‐dependent inhibitory effect on the release of IL‐1β induced by ox‐LDL. RT‐qPCR analysis revealed a reduction in the transcript levels of IL‐1β and IL‐18 with increasing doses of GI‐Y2 (Figure 6D,E). Similar trends of supernatant IL‐1β and IL‐18 were observed in GI‐Y2‐treated MPMs (Figure 6F–H). Western blot analysis showed a dose‐dependent reduction in the protein expression of GSDMD‐FL, and GSDMD‐N in ox‐LDL‐treated MPMs (Figure 6F,I,J). Likewise, GI‐Y2 inhibited the membrane expression of GSDMD‐N in a dose‐dependent manner (Figure 6K,L). To verify the clinical application potential of GI‐Y2, we collected human macrophages and observed that GI‐Y2 effectively inhibited the ox‐LDL‐induced IL‐1β released and the membrane aggregation of GSDMD‐N (Figure S6). These results demonstrated that GI‐Y2 inhibits ox‐LDL‐induced pyroptosis in cultured macrophages.

### 3.7 GI‐Y2 loaded in macrophage membrane‐coated nanoparticles alleviates atherosclerosis by targeting macrophages in atheromatous plaques

**FIGURE 7:**
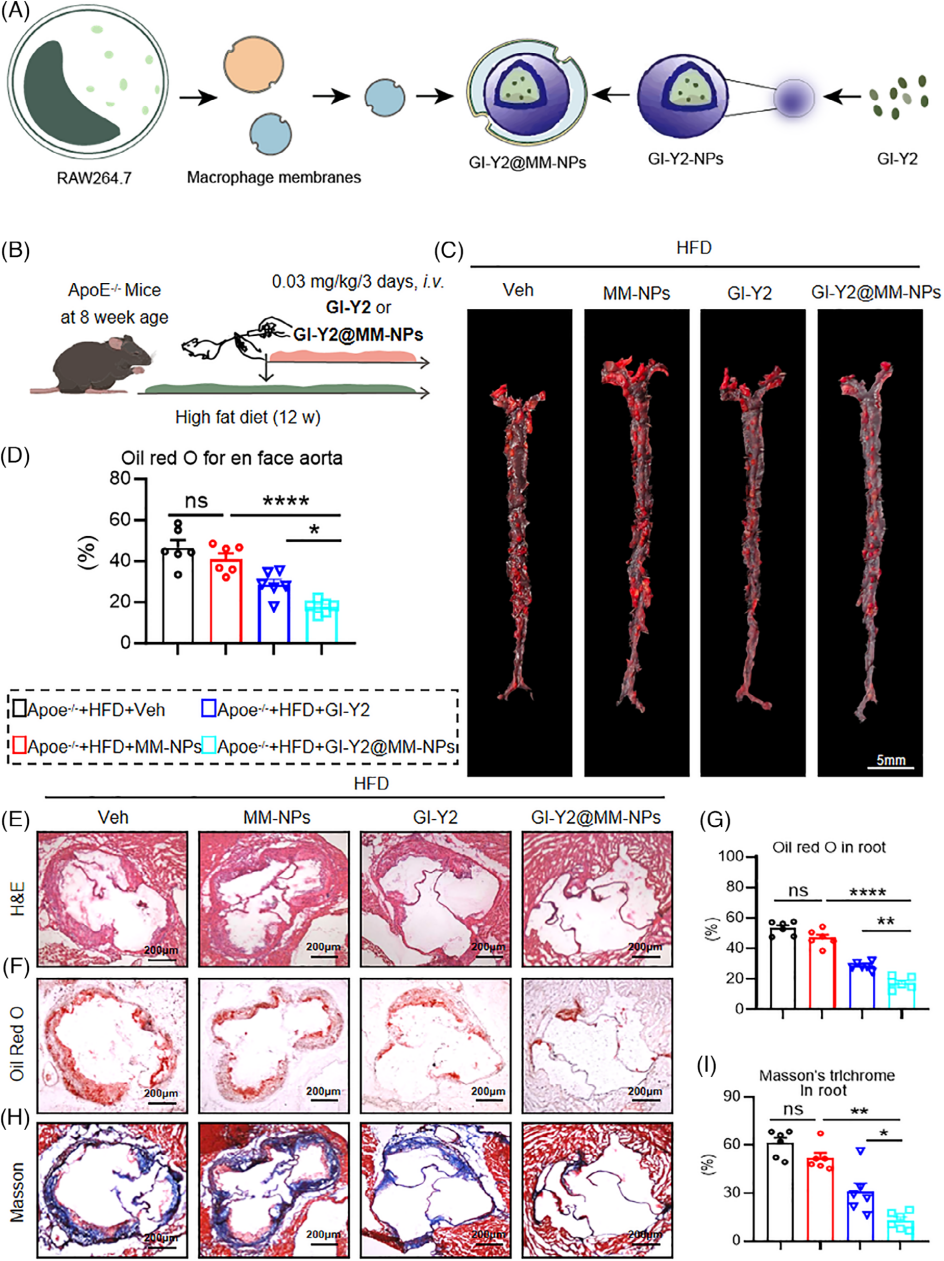
GI‐Y2 loaded in macrophage membrane‐coated nanoparticles alleviated atherosclerosis by targeting macrophages in atheromatous plaques. (A) A schematic diagram of the preparation method of GI‐Y2@MM‐NPs. MM‐NPs were produced by sonication of RAW264.7 cells. Subsequently, GI‐Y2 was encapsulated into MM‐NPs using poly(lactic acid‐glycolic acid) (PLGA) technology. (B) *ApoE* ^−/−^ mice were fed a HFD for 8 weeks, followed by the administration of GI‐Y2@MM‐NPs (0.03 mg/kg/3 days, *i.v*.) for an additional 4 weeks. (C, D) Oil Red O staining of the en face aorta (C) and quantification (D). (E) HE staining of the aortic root. (F, G) Oil Red O staining (F) of the aortic root and quantification of positive areas (G). (H, I) Masson's trichrome staining (H) of the aortic root and quantification of fibrotic areas (I). (*n* = 6, ns *p* > 0.05, * *p* < 0.05, ** *p *< 0.01, **** *p* < 0.0001).

GSDMD is principally distributed in macrophages during atherosclerosis.^13^ Macrophages in atheromatous plaques promote positive feedback to recruit additional inflammatory cells to these areas.^28^ To enhance the targeting of GI‐Y2 to macrophages in atheromatous plaques, we constructed macrophage membrane‐coated GI‐Y2 nanoparticles (GI‐Y2@MM‐NPs). MM‐NPs were produced by sonication of the RAW264.7 cell line (Figure S7A). GI‐Y2 was encapsulated into MM‐NPs using PLGA technology^29^ (Figure 7A). As shown in Figure S7B,C, the diameter of the GI‐Y2@MM‐NPs was between 200 and 300 nanometres. To confirm the ability of GI‐Y2@MM‐NPs to target atheromatous plaques, the photodynamic agent DilC18(5) (DiD) was applied to aortas harvested from mice. Using an ex vivo fluorescence bioimaging system, we found that the aorta tissues of the GI‐Y2@MM‐NP‐treated group exhibited stronger fluorescence than those of the GI‐Y2‐treated group (Figure S7D). The fluorescence images of F4/80 and DID in aortic sections also show that the group treated with GI‐Y2@MM‐NPs exhibited stronger overlapping fluorescence compared to the group treated with GI‐Y2 alone (Figure S7E).

We investigated the biosafety of GI‐Y2@MM‐NPs in vivo. Administration of GI‐Y2@MM‐NPs did not affect the body weight, haematologic system, hepatic function, renal function, or tissue structure of the brain, lung, liver, kidney or heart (Figure S8). *ApoE^−/−^* mice underwent an 8‐week HFD diet, subsequently receiving GI‐Y2@MM‐NPs (0.03 mg/kg/3 days, *i.v*.) for 4 additional weeks (Figure 7B). (To accommodate the requirements for intravenous administration of GI‐Y2@MM‐NPs, we adjusted the dosing frequency and cycle to achieve a comparable total dosage.) Oil Red O staining of the En face aortas demonstrated that GI‐Y2@MM‐NPs exhibited greater inhibition of atherosclerotic plaques than did GI‐Y2 (Figure 7C,D). Similarly, GI‐Y2@MM‐NPs were more effective than GI‐Y2 at reducing the lesion area (Figure 7E), lipid deposition (Figure 7F,G) and interstitial fibrosis (Figure 7H,I) in the aortic roots of HFD‐fed *ApoE^−/−^* mice. However, the administration of MM‐NPs alone did not exert a substantial impact on the formation of atherosclerotic plaques. The above results proved that GI‐Y2@MM‐NPs can target macrophages in atheromatous plaques and have a therapeutic effect on atherosclerosis.

## 4 DISCUSSION

Identifying and developing new inhibitors of GSDMD could be a promising strategy for treating pyroptosis‐mediated diseases. Compared with NLRP3 or Caspase1/4/5 inhibitors, GSDMD inhibitors simultaneously reduce pyroptosis induced by multiple inflammasome components, such as NLPR3 and inflammatory caspases.^5^, ^6^ In addition, unlike neutralizing antibodies against single inflammatory factors, GI‐Y2, which inhibits GSDMD‐N induced pore formation on the cell membrane, can simultaneously block the release of multiple pyroptotic cytokines, such as IL‐1β and IL‐18.^5^, ^6^ Several strategies have been applied to the development of GSDMD inhibitors, including rational design,^30^, ^31^ fluorogenic liposome leakage assay‐based high‐throughput screening^32^ and structure‐based virtual screening.^19^ Structure‐based virtual screening is used to identify the most promising compounds for drug discovery from databases through computational docking methods.^33^, ^34^ In our previous study, we revealed GI‐Y1 as an inhibitor of GSDMD via GSDMD‐N‐based virtual screening and confirmed the protective effect of GI‐Y1 on myocardial ischemia/reperfusion injury.^19^ In this study, we optimized the virtual screening method and screened out GI‐Y2 as another potential candidate using full‐length GSDMD as the template.

Currently, GSDMD inhibitors have been developed to reduce pyroptosis through different mechanisms, such as inhibiting the cleavage of GSDMD^35^ and reducing the cytomembrane oligomerization of GSDMD‐N.^31^, ^32^ The mechanisms of action of GSDMD inhibitors are mainly based on the amino acid binding sites of GSDMD. Among these GSDMD inhibitors, both disulfiram^32^ and necrosulfonamide^36^ bind to Cys191 of GSDMD, which is a crucial residue for the oligomerization of GSDMD‐N. Emodin interacts with the Trp415 and Leu290 residues of GSDMD, thereby inhibiting the cleavage of GSDMD.^35^ Here, we demonstrated that GI‐Y2 inhibits the binding between cytomembrane and GSDMD‐N to reduce pyroptosis. Our molecular docking and molecular dynamics (MD) simulations revealed that the top 10 residues contributing most significantly to the interaction with GI‐Y2 are Arg11, Arg10, Met1, Arg7, His176, Ile90, Arg174, Val440, Met86, and Gly2. Among these, the highest‐ranked residues, Arg10 and Arg11, form hydrogen bonds with GI‐Y2. Structural prediction analysis indicated that Arg11 does not play a significant role in NTD‐CTD domain interactions, lipid binding, or oligomerization.^27^ Furthermore, we discovered that GI‐Y2 directly interacts with GSDMD and specifically targets the Arg10 residue. The amino acid Arg10 is located within the α1 helix of the secondary structure, which is indispensable for the lipid‐binding function of GSDMD‐N.^27^ This structural relationship aligns with the mechanism by which GI‐Y2 exerts its pharmacological activity through inhibiting lipid binding and pyroptotic pore formation of GSDMD‐N. Although there are species differences in the Arg10 residue (with Arg10 predominantly found in homospecies),^27^ the α1 helix, where Arg10 resides, may represent a promising secondary structure for the development of GSDMD inhibitors. In contrast, GI‐Y1 binds to the Arg7 residue of GSDMD as previously reported in our earlier publication.^19^ Notably, Arg10 is predicted to play a dual role in lipid binding and as an interface between the N‐terminal and C‐terminal domains of GSDMD, whereas Arg7 is only involved in lipid binding.^27^ This functional distinction may account for the stronger inhibitory activity of GI‐Y2 on GSDMD compared to GI‐Y1.

Recently, we reported that the expression of GSDMD is increased in atherosclerosis and that GSDMD deficiency ameliorates atherosclerotic plaque formation.^13^ Many studies^15^, ^18^ have shown that GSDMD is a promising drug target for atherosclerosis therapy. Here, GI‐Y2 exhibited a therapeutic effect on the formation of atherosclerotic plaques. GI‐Y2 was administered to *ApoE^−/‐^* mice following a 6‐week period of HFD. This administered strategy was specifically employed to replicate therapeutic interventions in a clinical setting. Additionally, we have previously established that atherosclerosis develops prior to the initiation of inhibitor administration in HFD‐fed mice after 6 weeks, as reported in our earlier publication.^13^ Furthermore, using *Gsdmd* ^−/−^ *ApoE* ^−/−^ mice, we ascertained that the protective effect of GI‐Y2 against atherosclerosis is mainly achieved by targeting GSDMD. Our previous studies revealed that GSDMD is principally distributed in atherosclerotic macrophages and that macrophage‐derived GSDMD promotes aortic pyroptosis and atherosclerotic plaque formation in vivo.^13^ Here, we also demonstrated the inhibitory effect of GI‐Y2 on pyroptosis and macrophage infiltration during atherosclerosis. Additionally, GI‐Y2 inhibited ox‐LDL‐induced macrophage pyroptosis in cultured MPMs isolated from *ApoE* ^−/−^ mice.

Currently, some GSDMD inhibitors exhibit organ toxicity, such as the FDA‐approved disulfiram (DSF). DSF has been reported to reduce atherosclerosis in hyperlipidaemic mice,^37^ but the concomitant cardiotoxicity^38^ limits its use in CVD treatment. Although GI‐Y2 has no obvious side effects, the lack of targeting efficacy would cause unsatisfactory accumulation at the aortic plaques after systemic administration. Cell membrane‐coated nanoparticles (M‐NPs) have emerged as a promising drug delivery method.^39^, ^40^

M‐NPs possess not only the capability to deliver nanoparticles but also demonstrate the inherent biological functionalities of the cell membrane, which can target injured tissues.^41^ In CVDs, a precedent has been established for the utilization of M‐NPs in the treatment of coronary artery disease.^42^ Macrophages in atheromatous plaques promote positive feedback to recruit additional inflammatory cells to these areas.^28^ Given that GSDMD is principally distributed in macrophages during atherosclerosis,^13^ we constructed macrophage membrane‐coated GI‐Y2 nanoparticles (GI‐Y2@MM‐NPs) to enhance the targeting of GI‐Y2 to macrophages in atheromatous plaques. The membrane receptors of macrophages can target specific ligands and surface proteins.^43^, ^44^ Likewise, GI‐Y2@MM‐NPs targeted atherosclerotic plaques via relevant proteins on the membrane surface of macrophages. GI‐Y2@MM‐NPs can significantly reduce atherosclerotic plaque formation. The drug utilization rate of GI‐Y2@MM‐NPs is better than that of GI‐Y2, suggesting that GI‐Y2@MM‐NPs have precise targeting ability and in vivo stability.

Technologies regarding the size and electrochemical gradient of GSDMD pores have emerged nowadays. In the study of GSDMD inhibitors similar to GI‐Y2, such as disulfiram, researchers found that using disulfiram could significantly alleviate the pore formation of GSDMD in nanodiscs.^32^ Unfortunately, the study still does not provide information on the change in pore size. As for cryoelectron microscopy,^26^ but if we need to study the effect of GI‐Y2 on the electrochemical gradient of GSDMD protein, we would be affected by the impact of DMSO on liposomes. The difficulty in obtaining these images remains a limitation of our experiments, and we hope to improve the experimental conditions in subsequent research to obtain representative images.

In summary, we identified a novel GSDMD inhibitor, GI‐Y2, via structure‐based virtual screening and pharmacological validation. Mechanistically, GI‐Y2 directly interacts with GSDMD and targets the Arg10 residue to reduce the membrane binding of GSDMD‐N. Functionally, we revealed that GI‐Y2 inhibits the formation of atherosclerotic plaques by targeting GSDMD. Similarly, GI‐Y2 reduces pyroptosis and macrophage infiltration in atherosclerosis. Furthermore, we constructed macrophage membrane‐coated GI‐Y2 nanoparticles to enhance the targeting of GI‐Y2 to macrophages in atheromatous plaques and demonstrated its vascular protective effect in vivo. This work revealed a therapeutic drug for CVD that targets GSDMD and provided new insights for the study of GSDMD‐mediated pyroptosis.

## AUTHOR CONTRIBUTIONS

X.F., Z.C., R.S., K.Y., Z.T., and S.S. carried out the experiments. B.Y., J.H., and S.D. contributed to the literature search and study design. J.H., B.Y., and X.F. participated in the drafting of the article. X.F., X.C., W.Z., and W.H. contributed to data collection and analysis. B.Y., J.H., and S.D. contributed essential reagents or tools.

## CONFLICT OF INTEREST STATEMENT

The authors declare no conflicts of interest.

## Supporting information



Supporting Information

## Data Availability

All the data in this study are available upon reasonable request from the corresponding author.

## References

[1] Benjamin EJ , Muntner P , Alonso A , et al. Heart Disease and Stroke Statistics‐2019 Update: a report from the American Heart Association. Circulation. 2019;139(10):e56‐e528. 30700139 10.1161/CIR.0000000000000659

[2] Dickhout JG , Basseri S , Austin RC . Macrophage function and its impact on atherosclerotic lesion composition, progression, and stability: the good, the bad, and the ugly. Arterioscler Thromb Vasc Biol. 2008;28(8):1413‐1415. 18650503 10.1161/ATVBAHA.108.169144

[3] He X , Fan X , Bai B , et al. Pyroptosis is a critical immune‐inflammatory response involved in atherosclerosis. Pharmacol Res. 2021;165:105447. 33516832 10.1016/j.phrs.2021.105447

[4] Neels JG , Gollentz C , Chinetti G . Macrophage death in atherosclerosis: potential role in calcification. Front Immunol. 2023;14:1215612. 37469518 10.3389/fimmu.2023.1215612 PMC10352763

[5] Shi J , Zhao Y , Wang K , et al. Cleavage of GSDMD by inflammatory caspases determines pyroptotic cell death. Nature. 2015;526(7575):660‐665. 26375003 10.1038/nature15514

[6] Kayagaki N , Stowe IB , Lee BL , et al. Caspase‐11 cleaves gasdermin D for non‐canonical inflammasome signalling. Nature. 2015;526(7575):666‐671. 26375259 10.1038/nature15541

[7] Dai S , Ye B , Zhong L , et al. GSDMD mediates LPS‐induced septic myocardial dysfunction by regulating ROS‐dependent NLRP3 inflammasome activation. Front Cell Dev Biol. 2021;9:779432. 34820388 10.3389/fcell.2021.779432 PMC8606561

[8] Ye B , Shi X , Xu J , et al. Gasdermin D mediates doxorubicin‐induced cardiomyocyte pyroptosis and cardiotoxicity via directly binding to doxorubicin and changes in mitochondrial damage. Transl Res. 2022;248:36‐50. 35545198 10.1016/j.trsl.2022.05.001

[9] Han J , Dai S , Zhong L , et al. GSDMD (Gasdermin D) mediates pathological cardiac hypertrophy and generates a feed‐forward amplification cascade via mitochondria‐STING (stimulator of interferon genes) axis. Hypertension. 2022;79(11):2505‐2518. 36065823 10.1161/HYPERTENSIONAHA.122.20004

[10] Han B , Xu J , Shi X , et al. DL‐3‐n‐butylphthalide attenuates myocardial hypertrophy by targeting gasdermin D and inhibiting gasdermin D mediated inflammation. Front Pharmacol. 2021;12:688140. 34168567 10.3389/fphar.2021.688140 PMC8217660

[11] Fang Z , Wu G , Sheng J , et al. Gasdermin D affects aortic vascular smooth muscle cell pyroptosis and Ang II‐induced vascular remodeling. Heliyon. 2023;9(6):e16619. 37303505 10.1016/j.heliyon.2023.e16619 PMC10248119

[12] Ye B , Fan X , Fang Z , et al. Macrophage‐derived GSDMD promotes abdominal aortic aneurysm and aortic smooth muscle cells pyroptosis. Int Immunopharmacol. 2024;128:111554. 38262162 10.1016/j.intimp.2024.111554

[13] Fan X , Han J , Zhong L , et al. Macrophage‐derived GSDMD plays an essential role in atherosclerosis and cross talk between macrophages via the mitochondria‐STING‐IRF3/NF‐κB axis. Arterioscler Thromb Vasc Biol. 2024;44(6):1365‐1378. 38695170 10.1161/ATVBAHA.123.320612

[14] Jiang M , Sun X , Liu S , et al. Caspase‐11‐gasdermin D‐mediated pyroptosis is involved in the pathogenesis of atherosclerosis. Front Pharmacol. 2021;12:657486. 33981234 10.3389/fphar.2021.657486 PMC8109243

[15] Puylaert P , Van Praet M , Vaes F , et al. Gasdermin D deficiency limits the transition of atherosclerotic plaques to an inflammatory phenotype in ApoE knock‐out mice. Biomedicines. 2022;10(5):1171. 35625908 10.3390/biomedicines10051171 PMC9138554

[16] Huang B , Zou Z , Li Y , et al. Gasdermin D‐mediated pyroptosis promotes the development of atherosclerosis. Lab Invest. 2024;104(4):100337. 38266921 10.1016/j.labinv.2024.100337

[17] Hsu CC , Fidler TP , Kanter JE , et al. Hematopoietic NLRP3 and AIM2 inflammasomes promote diabetes‐accelerated atherosclerosis, but increased necrosis is independent of pyroptosis. Diabetes. 2023;72(7):999‐1011. 37083999 10.2337/db22-0962 PMC10281813

[18] Opoku E , Traughber CA , Zhang D , et al. Gasdermin D mediates inflammation‐induced defects in reverse cholesterol transport and promotes atherosclerosis. Front Cell Dev Biol. 2021;9:715211. 34395445 10.3389/fcell.2021.715211 PMC8355565

[19] Zhong L , Han J , Fan X , et al. Novel GSDMD inhibitor GI‐Y1 protects heart against pyroptosis and ischemia/reperfusion injury by blocking pyroptotic pore formation. Basic Res Cardiol. 2023;118(1):40. 37782407 10.1007/s00395-023-01010-4

[20] Le Guilloux V , Schmidtke P , Tuffery P . Fpocket: an open source platform for ligand pocket detection. BMC Bioinf. 2009;10:168. 10.1186/1471-2105-10-168 PMC2700099 19486540

[21] Ravindranath PA , Forli S , Goodsell DS , Olson AJ , Sanner MF . AutoDockFR: advances in protein‐ligand docking with explicitly specified binding site flexibility. PLoS Comput Biol. 2015;11(12):e1004586. 26629955 10.1371/journal.pcbi.1004586 PMC4667975

[22] Han J , Shi X , Zheng Z , et al. Schisandrin B protects against angiotensin II‐induced endotheliocyte deficits by targeting Keap1 and activating Nrf2 pathway. Drug Des Devel Ther. 2018;12:3985‐3997. 10.2147/DDDT.S184245 PMC6255115 30538426

[23] Fu W , Zhang M , Liao J , et al. Discovery of a novel androgen receptor antagonist manifesting evidence to disrupt the dimerization of the ligand‐binding domain via attenuating the hydrogen‐bonding network between the two monomers. J Med Chem. 2021;64(23):17221‐17238. 34809430 10.1021/acs.jmedchem.1c01287

[24] Lomenick B , Hao R , Jonai N , et al. Target identification using drug affinity responsive target stability (DARTS). Proc Natl Acad Sci USA. 2009;106(51):21984‐21989. 19995983 10.1073/pnas.0910040106 PMC2789755

[25] Han J , Shi X , Xu J , et al. DL‐3‐n‐butylphthalide prevents oxidative stress and atherosclerosis by targeting Keap‐1 and inhibiting Keap‐1/Nrf‐2 interaction. Eur J Pharm Sci. 2022;172:106164. 35259495 10.1016/j.ejps.2022.106164

[26] Xia S , Zhang Z , Magupalli VG , et al. Gasdermin D pore structure reveals preferential release of mature interleukin‐1. Nature. 2021;593(7860):607‐611. 33883744 10.1038/s41586-021-03478-3 PMC8588876

[27] Liu Z , Wang C , Yang J , et al. Crystal structures of the full‐length murine and human gasdermin D reveal mechanisms of autoinhibition, lipid binding, and oligomerization. Immunity. 2019;51(1):43‐49. e44. 31097341 10.1016/j.immuni.2019.04.017 PMC6640092

[28] Moore KJ , Sheedy FJ , Fisher EA . Macrophages in atherosclerosis: a dynamic balance. Nat Rev Immunol. 2013;13(10):709‐721. 23995626 10.1038/nri3520 PMC4357520

[29] Ou Z , Zhong H , Zhang L , et al. Macrophage Membrane‐coated nanoparticles alleviate hepatic ischemia‐reperfusion injury caused by orthotopic liver transplantation by neutralizing endotoxin. Int J Nanomedicine. 2020;15:4125‐4138. 32606668 10.2147/IJN.S253125 PMC7296981

[30] Yang J , Liu Z , Wang C , et al. Mechanism of gasdermin D recognition by inflammatory caspases and their inhibition by a gasdermin D‐derived peptide inhibitor. Proc Natl Acad Sci USA. 2018;115(26):6792‐6797. 29891674 10.1073/pnas.1800562115 PMC6042100

[31] Chen Y , Luo R , Li J , et al. Intrinsic radical species scavenging activities of tea polyphenols nanoparticles block pyroptosis in endotoxin‐induced sepsis. ACS nano. 2022;16(2):2429‐2441. 35133795 10.1021/acsnano.1c08913

[32] Hu JJ , Liu X , Xia S , et al. FDA‐approved disulfiram inhibits pyroptosis by blocking gasdermin D pore formation. Nat Immunol. 2020;21(7):736‐745. 32367036 10.1038/s41590-020-0669-6 PMC7316630

[33] Yang X , Wang Y , Byrne R , Schneider G , Yang S . Concepts of artificial intelligence for computer‐assisted drug discovery. Chem Rev. 2019;119(18):10520‐10594. 31294972 10.1021/acs.chemrev.8b00728

[34] Kitchen DB , Decornez H , Furr JR , Bajorath J . Docking and scoring in virtual screening for drug discovery: methods and applications. Nat Rev Drug Discovery. 2004;3(11):935‐949. 15520816 10.1038/nrd1549

[35] Dai S , Chen Y , Fan X , et al. Emodin attenuates cardiomyocyte pyroptosis in doxorubicin‐induced cardiotoxicity by directly binding to GSDMD. Phytomedicine. 2023;121:155105. 37801893 10.1016/j.phymed.2023.155105

[36] Rathkey JK , Zhao J , Liu Z , et al. Chemical disruption of the pyroptotic pore‐forming protein gasdermin D inhibits inflammatory cell death and sepsis. Sci Immunol. 2018;3(26):eaat2738. 30143556 10.1126/sciimmunol.aat2738 PMC6462819

[37] Traughber CA , Timinski K , Prince A , et al. Disulfiram reduces atherosclerosis and enhances efferocytosis, autophagy, and atheroprotective gut microbiota in hyperlipidemic mice. Biorxiv. 2023. 10.1161/JAHA.123.033881 PMC11262521 38563369

[38] Li Y , Shen J , Fang M , et al. The promising antitumour drug disulfiram inhibits viability and induces apoptosis in cardiomyocytes. Biomed Pharmacother. 2018;108:1062‐1069. 30372806 10.1016/j.biopha.2018.09.123

[39] Fang RH , Gao W , Zhang L . Targeting drugs to tumours using cell membrane‐coated nanoparticles. Nat Rev Clin Oncol. 2023;20(1):33‐48. 36307534 10.1038/s41571-022-00699-x

[40] Krishnan N , Jiang Y , Zhou J , et al. A modular approach to enhancing cell membrane‐coated nanoparticle functionality using genetic engineering. Nat Nanotechnol. 2024;19(3):345‐353. 37903891 10.1038/s41565-023-01533-w PMC10954421

[41] Parodi A , Quattrocchi N , van de Ven AL , et al. Synthetic nanoparticles functionalized with biomimetic leukocyte membranes possess cell‐like functions. Nat Nanotechnol. 2013;8(1):61‐68. 23241654 10.1038/nnano.2012.212 PMC3751189

[42] Gao C , Huang Q , Liu C , et al. Treatment of atherosclerosis by macrophage‐biomimetic nanoparticles via targeted pharmacotherapy and sequestration of proinflammatory cytokines. Nat Commun. 2020;11(1):2622. 32457361 10.1038/s41467-020-16439-7 PMC7251120

[43] Narain A , Asawa S , Chhabria V , Patil‐Sen Y . Cell membrane coated nanoparticles: next‐generation therapeutics. Nanomedicine (Lond). 2017;12(21):2677‐2692. 28965474 10.2217/nnm-2017-0225

[44] Wang Z , Li J , Cho J , Malik AB . Prevention of vascular inflammation by nanoparticle targeting of adherent neutrophils. Nat Nanotechnol. 2014;9(3):204‐210. 24561355 10.1038/nnano.2014.17 PMC4100792

