# Supplementary material for: The novel GSDMD inhibitor GI‐Y2 exerts antipyroptotic effects to reduce atherosclerosis
Source: Clin Transl Med. 2025 Mar 5;15(3):e70263. doi: 10.1002/ctm2.70263 (PMC11882392; doi:10.1002/ctm2.70263)
Supplement: Supplementary file 1 — Supporting Information [file CTM2-15-e70263-s001.docx]

***Supplemental Material***

**The novel GSDMD inhibitor GI-Y2 exerts antipyroptotic effects to reduce atherosclerosis**

Supplementary Information:

1. Supplementary Figure S1-8
2. Supplementary Table S1

***Supplementary Figures:***

**
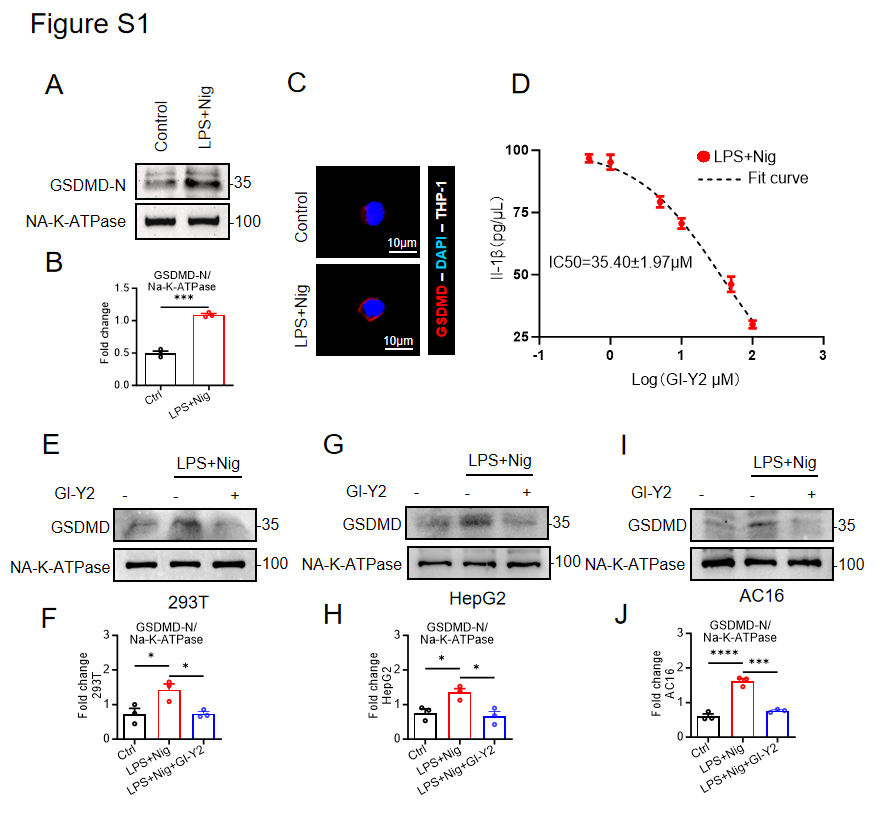
**

**Figure S1 A-C** THP-1 cells were pretreated with PMA and then stimulated with LPS+nigericin (LN; 1 μg/ml, 4 h for LPS; 10 μM, 30 min for nigericin). (**A**) Expression of GSDMD-N in the THP-1cell membrane and (**B**) quantification (n=3). (**C**) Representative immunofluorescence images of THP-1 cells (n=3). **D** Dose response curve of GI-Y2 in Il-1β Elisa assay. (n=3). The mean ± s.e.m. is shown. **E-J** 293T, HepG2 and AC16 cells were pretreated with GI-Y2 (20 μM, 1 h) and then stimulated with LPS+nigericin (LN; 1 μg/ml, 4 h for LPS; 10 μM, 30 min for nigericin) (n=3). Expression of GSDMD-N in the 293T cell membrane (**E**) and quantification (**F**). Expression of GSDMD-N in the HepG2 cell membrane (**G**) and quantification (**H**). Expression of GSDMD-N in the AC16 cell membrane (**I**) and quantification (**J**). * P <0.05, *** P < 0.001, **** P < 0.0001.

**
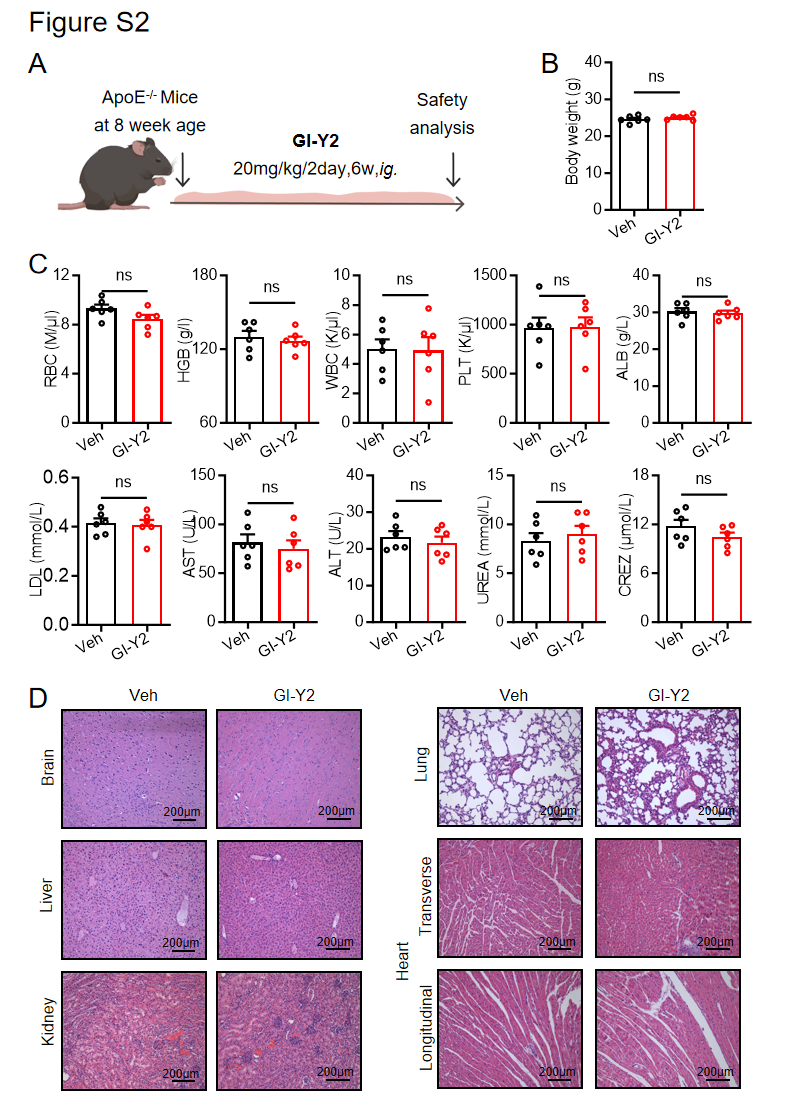
**

**Figure S2 A** Mice were treated with GI-Y2 (20 mg/kg/day, i.g.) or vehicle for 6 weeks and euthanized under sodium pentobarbital anesthesia. Blood was collected for further analysis. **B** Body weight at 14 weeks. **C** A hemogram was conducted to assess parameters such as RBCs, HGB, WBCs and PLTs. Liver function was assessed by measuring plasma ALB, LDL, AST and ALT levels. Kidney function was assessed by plasma UREA and CREZ levels. All tests were assessed by an automatic biochemical analyzer (AU480, Beckman Coulter, America) (n=6, ns represents P>0.05). **D** H&E staining of major organs.

**
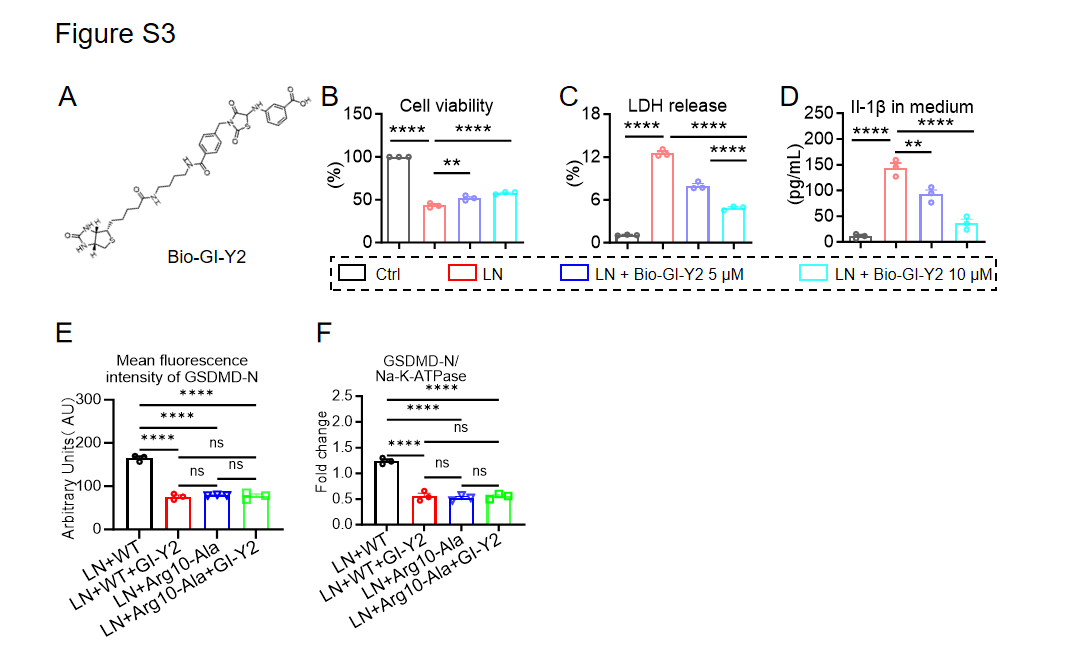
**

**Figure S3 A** Molecular formula of Bio-GI-Y2. **B-D** THP-1 cells stimulated with PMA were pretreated with Bio-GI-Y2 (5 μM, 1 h; 10 μM, 1 h) and then stimulated with LPS+nigericin (LN; 1 μg/ml, 4 h for LPS; 10 μM, 30 min for nigericin). Cell viability (**B**), LDH release (**C**) and IL-1β release (**D**) were measured. **E-F**: Quantification of immunofluorescence staining of GSDMD-N in HEK/293T membranes (**E**). Quantification of GSDMD-N expression in HEK293T cell membranes (**F**). (n=3, ** P<0.01, **** P<0.0001).


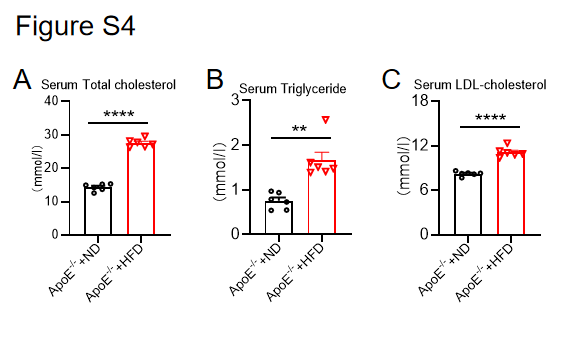


**Figure S4 A-C** Total cholesterol (**A**), triglyceride (**B**) and LDL-cholesterol (**C**) levels in the serum of *ApoE*^-/-^ mice fed a ND or HFD were detected by an automatic biochemical analyzer (AU480, Beckman Coulter, USA). (n=6, ** P<0.01, **** P<0.0001).


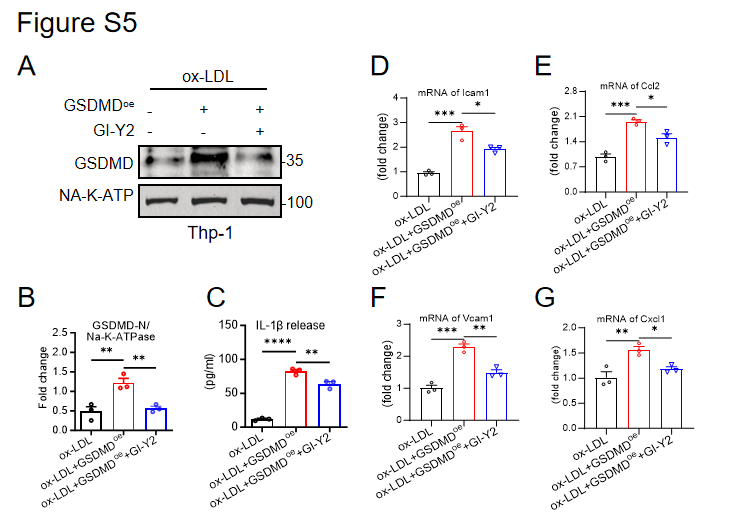


**Figure S5 A-G** MPMs were transfected with GSDMD plasmid, then pretreated with GI-Y2 (20 μM, 1 h) or vehicle (DMSO, 1 h) and stimulated with ox-LDL (50 μg/ml, 24 h). (**A**) Expression of GSDMD-N in the MPMs membrane and (**B**) quantification (n=3). (**C**) ELISA was used to measure the release of IL-1β in ox-LDL-treated MPMs (n=3). **D-E** The mRNA levels of Icam1 (**D**), Ccl2 (**E**), Vcam1 (**F**) and Cxcl (**G**) in MPMs (n=3). * P <0.05, ** P < 0.01, *** P < 0.001.


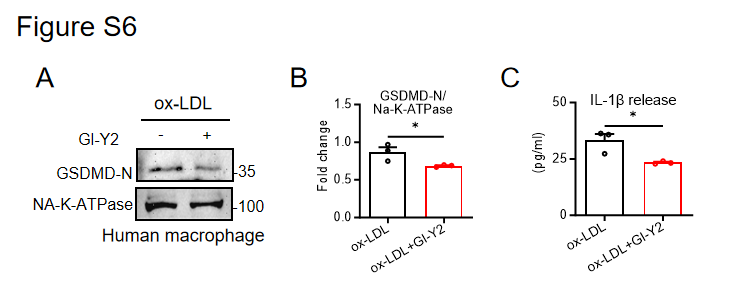


**Figure S6 A-C** Human macrophages were pretreated with GI-Y2 (20 μM, 1 h) or vehicle (DMSO, 1 h) and stimulated with ox-LDL (50 μg/ml, 24 h). (**A**) Expression of GSDMD-N in the Human macrophage membrane and (**B**) quantification (n=3).. (**C**) ELISA was used to measure the release of IL-1β in ox-LDL-treated Human macrophages (n=3). * P <0.05, ** P < 0.01.


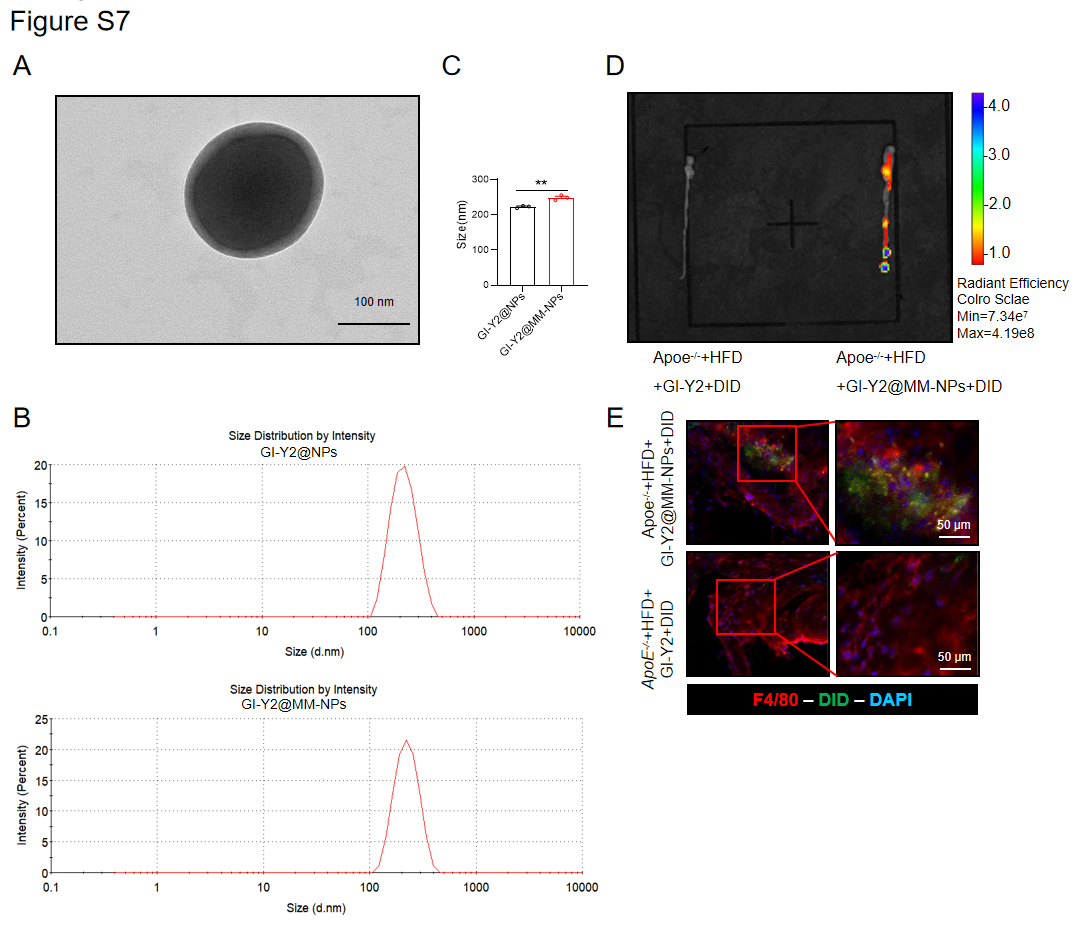


**Figure S7 A** Representative TEM image of [GI-Y2@MM-NPs (scale bar = 100 nm).](mailto:GI-Y2@MM-NPs._x0005_) **B‒C** Particle sizes of the GI-Y2-NPs and GI-Y2@MM-NPs were analyzed by a laser nanometer particle size analyzer (Zetasizer Nano ZS, Malvern, UK, n=3). **D-E** *ApoE*^-/-^ mice were fed a HFD for 8 weeks, followed by the administration of GI-Y2+DiD (0.03 mg/kg/once, i.v.) and GI-Y2@MM-NPs+DID (0.03 mg/kg/once, i.v.). Representative fluorescence images showed the accumulation of GI-Y2@MM-NPs with DiD in atherosclerotic tissue (**D**). Representative immunofluorescence images showing the colocalization of F4/80 and DID in aortic sections (**E)**.


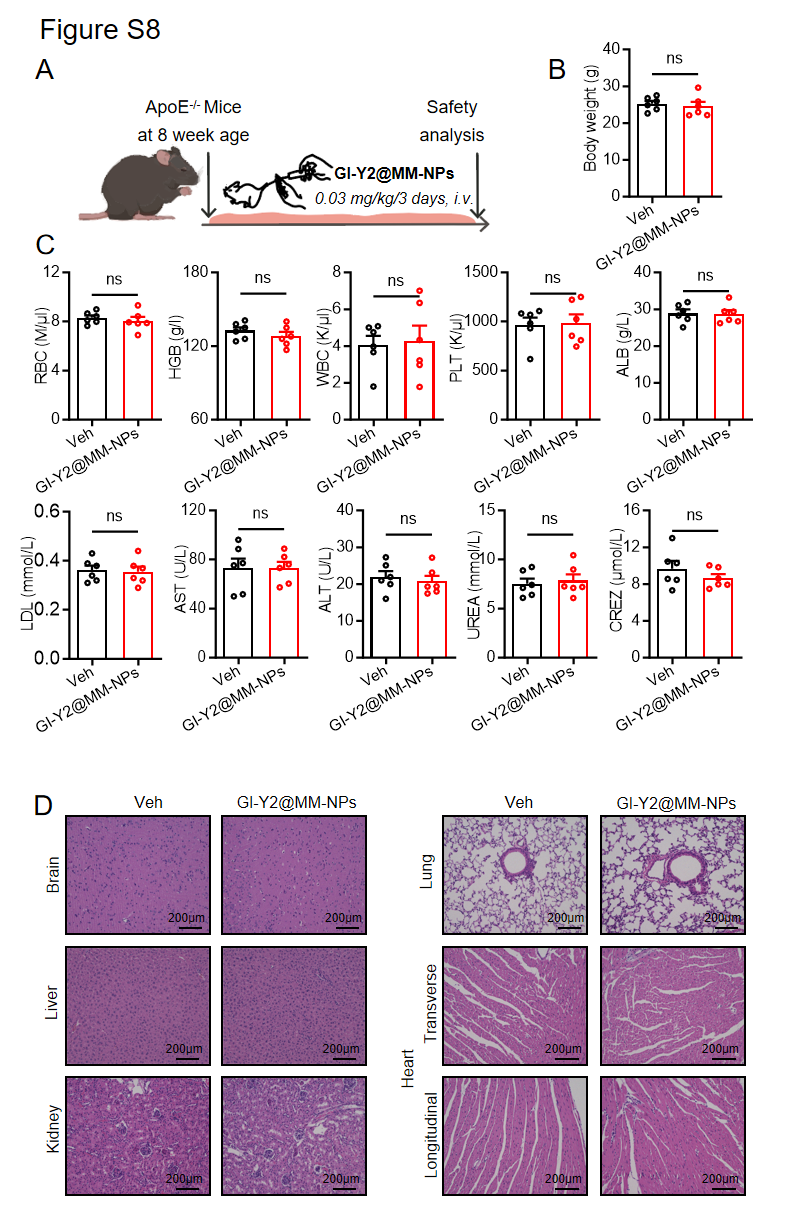


**Figure S8 A** Mice were treated with GI-Y2@MM-NPs (0.03 mg / kg / 3 day, *i.v.*) or vehicle for 4 weeks and euthanized under sodium pentobarbital anesthesia. Blood was collected for further analysis. **B** Body weight at 12 weeks. **C** A hemogram was conducted to assess parameters such as RBCs, HGB, WBCs and PLTs. Liver function was assessed by measuring plasma ALB, LDL, AST and ALT levels. Kidney function was assessed by plasma UREA and CREZ levels. All tests were assessed by an automatic biochemical analyzer (AU480, Beckman Coulter, America) (n=6, ns represents P>0.05). **D** H&E staining of major organs.

***Supplementary Tables:***

**Table S1: Primer sequences for qPCR**

| **Gene** | **Species** | **Sequence(Forward)** | **Sequence(Reverse)** |
| --- | --- | --- | --- |
| *Gsdmd* | Mouse | ACTGAGGTCCACAGCCAAGAGG | GCCACTCGGAATGCCAGGATG |
| *β-actin* | Mouse | CTACCTCATGAAGATCCTGACC | CACAGCTTCTCTTTGATGTCAC |
| *Il-1β* | Mouse | TCGCAGCAGCACATCAACAAGAG | AGGTCCACGGGAAAGACACAGG |
| *Il-18* | Mouse | CAAAGTGCCAGTGAACCCCAGAC | ACAGAGAGGGTCACAGCCAGTC |
| *Cxcl* | Mouse | GCTGGGATTCACCTCAAGAACATC | GTGTGGCTATGACTTCGGTTTGG |
| *Ccl2* | Mouse | CACTCACCTGCTGCTACTCATTC | GCTTCTTTGGGACACCTGCTG |
| *Vcam1* | Mouse | GCCACCCTCACCTTAATTGCTATG | AATTCCACTTCTGCTTTGTCTCTCC |
| *Icam1* | Mouse | TGGAGACGCAGAGGACCTTAAC | CGACGCCGCTCAGAAGAAC |
